# Hyperglycemia induces gastric carcinoma proliferation and migration via the *Pin1/BRD4* pathway

**DOI:** 10.1038/s41420-022-01030-4

**Published:** 2022-04-23

**Authors:** Jianjian Yu, Dan Hu, Laicheng Wang, Zongcheng Fan, Changsheng Xu, Yunchai Lin, Xin Chen, Jinxiu Lin, Feng Peng

**Affiliations:** 1 Department of Cardiology, the First Affiliated Hospital, Fujian Medical University, Fuzhou, 350005 Fujian, China; 2 Department of Pathology, Fujian Medical University Cancer Hospital & Fujian Cancer Hospital, Fuzhou, 350011 Fujian, China; 3 Fujian Provincial Institute of Hypertension, the First Affiliated Hospital, Fujian Medical University, Fuzhou, 350005 Fujian, China

**Keywords:** Cancer metabolism, Oncogenes

## Abstract

Diabetes is a potential risk factor for gastric cancer (GC). *Pin1*, a peptidyl–prolyl cis/trans isomerase, promotes GC cell proliferation and migration. The role and underlying mechanism of the *Pin1/BRD4* axis in hyperglycemia-induced proliferation and migration of GC cells were analyzed in vivo and in vitro. Proliferation and migration of GC cells were measured; Pin1 and BRD4 expression of the cell cycle were determined. *Pin1* and *BRD4* were downregulated by transfecting Pin1 shRNA lentivirus into GC cells and JQ1-intervention GC cells. Tumor formation and lung metastasis were assessed in vivo. Inhibition of *Pin1* and *BRD4* significantly suppressed high-glucose (HG)-induced GC cell proliferation and migration. HG enhanced G1/S cell-cycle transition, associated with increased Pin1 and BRD4 expression. Silencing *Pin1* significantly downregulated the expression of BRD4 and NAP1L1 and upregulated that of P21 in GC cells. In vivo studies indicated that hyperglycemia promotes tumor growth and lung metastasis by inducing Pin1 and BRD4 expression. Thus, *Pin1/BRD4* plays an important role in hyperglycemia-promoted tumor growth. The significance of these findings toward improved prognosis of diabetic patients with GC cannot be underestimated.

## Introduction

Gastric cancer (GC) remains the third most prevalent cause of cancer-related death worldwide, and is a huge clinical and societal burden [1]. Epidemiological studies have demonstrated that diabetes may be a risk factor for GC [2]. Although a high-glucose (HG) microenvironment may be a factor linking diabetes and cancer, the role of hyperglycemia in GC progression and the potential molecular mechanisms underlying this process remain unclear.

We previously reported that the prognosis of patients with cancer is significantly worsened by hyperglycemia, which promotes tumor cell proliferation and metastasis, and that glucose-lowering therapy significantly extends overall survival [3–6]. We have therefore investigated the molecular mechanisms underlying the induction of cancer cell proliferation by HG in order to screen and identify new targets that are anti-abnormal cancer cell proliferation, which may lead to a therapeutic breakthrough for comorbid diabetes and cancer.

The peptidyl–prolyl cis/trans isomerase (PPIase) *Pin1* is composed of 163 amino acids, including a nuclear localization signal and two main functional domains [7]. It is a uniquely phosphorylation-dependent PPIase, which specifically recognizes the pSer/Thr–Pro motifs of many target proteins, and facilitates their cis–trans isomerization to regulate their stability, subcellular localization, and transcriptional activity [8, 9]. Abnormal regulation of *Pin1* exerts a profound effect on cell fate, and is therefore associated with the development of various diseases, tumorigenesis, and tumor development [10]. Many studies have indicated that Pin1 overexpression is significantly correlated with poor cancer prognoses, the underlying mechanism of which is associated with the promotion of cancer cell proliferation and migration by *Pin1* [11, 12]. Our previous studies have indicated that *Pin1* promotes inflammatory responses and oxidative stress, and participates in hyperglycemia-induced inflammation. *Pin1* also plays an important role in HG-induced cell proliferation and migration [13].

*BRD4* is a typical bromodomain and extraterminal domain (BET) protein family member. It plays a key role in gene regulation, DNA damage, cell cycle, and cell proliferation, and has been implicated in various cancers due to its association with transcriptional activation, which is accomplished by binding to chromatin via identification of acetylated histone proteins [14–17]. BET bromodomain inhibitor JQ1 binds to the acetyl–lysine-binding pocket of *BRD4* to inhabit BRD4 expression [18]. Previously, we revealed that BRD4 indirectly acts on the 3′-UTR of P21 mRNA through the regulation of miR-106b-5p, which downregulates P21 expression, thus inhibiting cell proliferation [19]. Recent studies have indicated that Pin1 enhances BRD4 stability via direct binding and by promoting BRD4 and CDK9 interaction, thereby increasing its transcriptional activity as well [20]. These results showed that BRD4 is a Pin1-targeting protein that interacts with it to regulate cell proliferation. However, whether hyperglycemia promotes high Pin1 expression in tumor tissues, leading to increased BRD4 expression and tumor cell proliferation, remains unclear.

Nucleosome assembly protein 1-like 1 (*NAP1L1*), which is widely expressed in various tissues and organs, participates in DNA replication and nucleosome assembly, and plays an important role in chromatin formation and transcriptional regulation [21, 22]. Some studies have found that *NAP1L1*, which is closely associated with cell growth, is significantly increased in proliferative cells [23]. It can inhibit the expression of P21 via the *AKT* signaling pathway, resulting in abnormal cell proliferation [24]. Although many studies have discussed the importance of *NAP1L1*, its role in hyperglycemia-induced proliferation and migration of gastric cancer cells remains undocumented.

This study explored hyperglycemia-induced proliferation and migration of GC cells, and the underlying involvement of the *Pin1/BRD4* signal pathway, both in vitro and in vivo. Its results may provide a theoretical basis for the screening and identification of new targets for antidiabetic tumor drugs.

## Results

### Effects of silencing *Pin1* or *BRD4* on HG-induced GC cell proliferation and migration

In order to elucidate the effect of *Pin1* and *BRD4* on HG-induced GC cell proliferation and migration, GC cells were transfected with ShPin1 lentivirus to silence *Pin1*, while *BRD4* was inhibited using JQ1. Relative mRNA expression levels of Pin1 in AGS, HGC27, and MKN45 cells, separately transfected with three shPin1 and one Sh-NC lentiviral vectors, were investigated using qRT-PCR. Notably, the results showed that *Pin1* in the Shpin1#1 group was markedly inhibited, while inhibition efficiency in the three cell types was increased more than threefold (Fig. S1a). Next, we used a ShRNA#1 lentivirus vector to knock down *Pin1*, and flow-cytometric sorting to screen transfected cells (Fig. S1b). Following preincubation of GC cells with JQ1 (10^−6^–10^−8 ^M) for 1 h and incubation with HG for 72 h, immunoblots showed that JQ1 downregulated BRD4 protein levels in a concentration-dependent manner (Fig. S2). In further experiments, JQ1 (10^−6 ^M) was used to inhibit BRD4 expression.

**Fig. 1:**
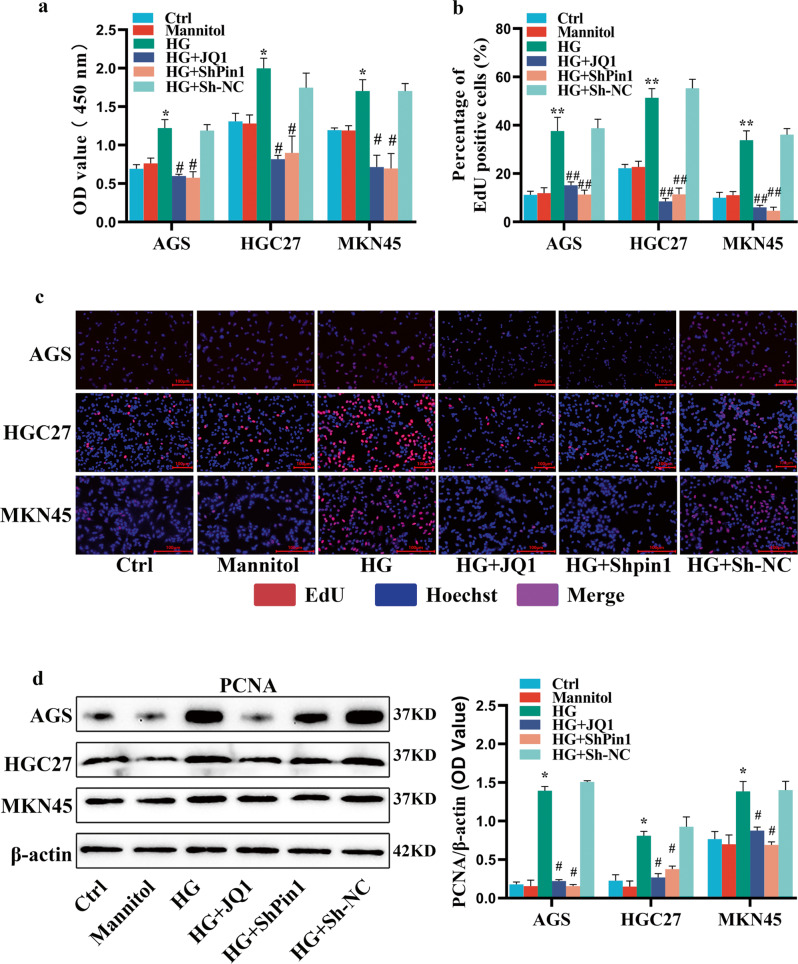
Effect of silencing *Pin1* or *BRD4* on proliferation of GC cells induced by HG. **a** CCK-8 assay was used to measure proliferation of GC cells. Cells were infected with lentivirus vector of ShPin1 or Sh-NC or were pretreated with JQ1 (10^−6 ^M) for 1 h and incubated with HG for 72 h. **b**, **c** GC cell proliferation was measured using EdU assays. EdU (red) and nuclei (blue, Hoechst) indicate proliferation. Scale bars, 100 μm. **d** PCNA proteins were extracted for WB. β-actin represents loading control. Values are mean ± SEM. ^*^P < 0.05 vs. Ctrl; ^**^P < 0.01 vs. Ctrl; ^#^P < 0.05 vs. HG; ^*##*^*P* < 0.01 vs. HG. All the experiments were performed 3 times.

CCK-8 analysis indicated that, compared with the ctrl group, HG facilitated the proliferation of AGS, HGC27, and MKN45 cells. Furthermore, the proliferation of GC cells promoted by HG was abrogated by the silencing of *Pin1* or inhibition of *BRD4* (Fig. 1a). The EdU incorporation assay confirmed these proliferation results (Fig. 1b, c). Meanwhile, WB indicated that PCNA expression in the HG group was enhanced. Silencing the *Pin1* or *BRD4* inhibited the increase in PCNA expression induced by HG (Fig. 1d).

**Fig. 2:**
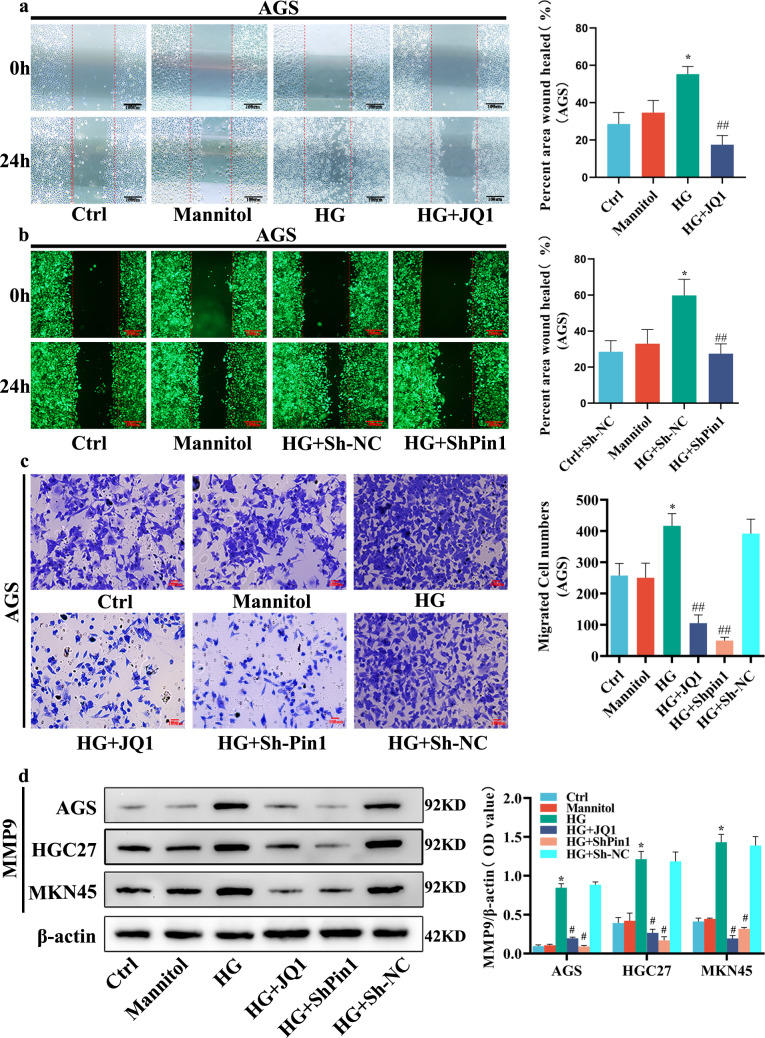
Effect of silencing *Pin1* or *BRD4* on migration of GC cells induced by HG. **a** Wound-healing assay was used to measure migration of AGS. AGS was treated with JQ1 (10^−6 ^M) and incubated with HG for 24 h. Scale bars, 100 μm. Values are mean ± SEM. ^*^P < 0.05 vs. Ctrl; ^*##*^*P* < 0.01 vs. HG. **b** Wound-healing assay of AGS transfected with the lentivirus vector of ShPin1 or Sh-NC. Scale bars, 100 μm. Values are mean ± SEM. ^*^P < 0.05 vs. Ctrl+Sh*-*NC; ^*##*^*P* < 0.01 vs. HG + Sh-NC. **c** Transwell assays were used to measure migration of AGS. Scale bars, 100 μm. Values are mean ± SEM. ^*^P < 0.05 vs. Ctrl; ^*##*^*P* < 0.01 vs. HG. **d** MMP9 proteins were extracted from cells for WB. β-actin served as the loading control. Values are mean ± SEM. ^*^P < 0.05 vs. Ctrl; ^*#*^*P* < 0.05 vs. HG. All the experiments were performed 3 times.

The migration ability of GC cells under HG conditions was assessed using wound-healing and Transwell assays. GC cell migration was markedly increased by HG, compared with the ctrl group, whereas silencing *Pin1* or *BRD4* significantly suppressed this increase (Fig. 2a, b and S3a, b). Consistent with this result, Transwell experiments demonstrated that GC cell migration induced by HG was significantly reduced by the downregulation of *Pin1* or *BRD4* (Fig. 2c and S3c, d). MMP9 expression, assessed via WB, further supported this result (Fig. 2d).

### *Pin1/BRD4* mediates HG-induced G1/S transition in GC cells

**Fig. 3:**
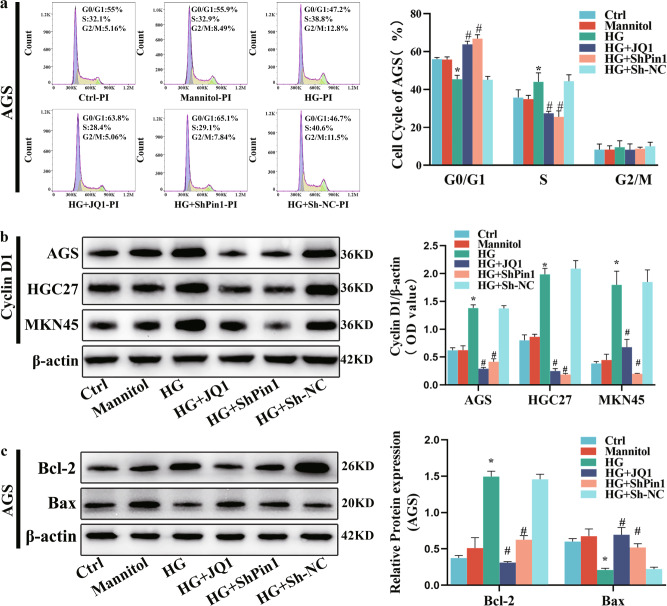
Effect of downregulation of *Pin1* or *BRD4* on HG-induced cell cycle. GC cells were infected with ShPin1 or Sh-NC lentivirus vectors or were treated with JQ1 (10^−6 ^M) and then incubated with HG for 72 h. **a** Subsequently, cells were fixed, stained with propidium iodide, and each group’s cell-cycle profile determined via flow cytometry. **b** Cyclin D1 proteins were extracted from cells for WB; β-actin represents loading control. **c** Bcl-2 and Bax proteins were extracted for WB; β-actin represents the loading control. Values are mean ± SEM. ^*^P < 0.05 vs. Ctrl; ^*#*^*P* < 0.05 vs^.^ HG; ^*##*^*P* < 0.01 vs. HG. All the experiments were performed 3 times.

Dysregulation of the cell cycle, resulting in aberrant cellular proliferation, is a hallmark of tumor cells. To explore whether HG affects the regulation of the cell-cycle progression by influencing the *Pin1/BRD4* axis, thereby promoting cellular proliferation, we analyzed cell cycle using flow cytometry. Our results showed that HG significantly promoted G1/S transition of GC cells. Compared with that in the ctrl group, the number of GC cells in the G0/G1 phase in the HG group was significantly reduced, while the number of GC cells in its S phase was significantly increased, with no significant difference in the G2/M phase. Inhibition of *Pin1* or *BRD4* significantly downregulated G1/S-phase transformation induced by HG (Fig. 3a and S4a, b). Next, we examined expression levels of cyclin D1, Bcl-2, and Bax via WB. These results demonstrated that HG markedly increased the expression of cyclin D1 and Bcl-2 and decreased Bax protein expression in GC cells, while downregulating the expression of Pin1 or BRD4 decreased cyclin D1 and Bcl-2 and increased Bax expression (Fig. 3b, c and S5).

### *Pin1/BRD4* axis regulates *P21* by targeting *NAP1L1*

**Fig. 4:**
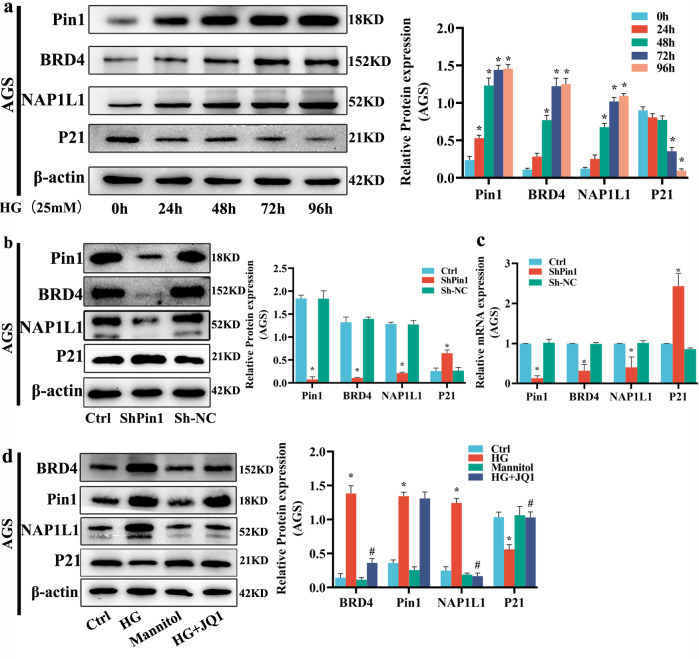
*Pin1/BRD4* axis regulates P21 by targeting NAP1L1. **a** AGS cells were treated with HG for 24, 48, 72, and 96 h. Pin1, BRD4, NAP1L1 and P21 protein expression was detected by WB; β-actin represents the loading control. Values are mean ± SEM; ^*^P < 0.05 vs. 0 h. **b**, **c** AGS was infected with lentivirus vector of ShPin1 or Sh-NC and incubated with HG for 72 h. Subsequently, Pin1, BRD4, NAP1L1, and P21 protein or mRNA expression were detected via WB and qRT-PCR; β-actin represents the loading control. Values are mean ± SEM; ^*^P < 0.05 vs^.^ Ctrl. **d** AGS was pretreated with JQ1 (10^−6^ M) for 1 h and incubated with HG for 72 h. Pin1, BRD4, NAP1L1, and P21 protein expression were detected by WB, β-actin represents the loading control. Values are mean ± SEM; ^*^P < 0.05 vs. Ctrl; ^*#*^*P* < 0.05 vs. HG. All the experiments were performed 3 times.

To explore the mechanism underlying the role of *Pin1/BRD4* axis in the proliferation and migration of GC cells induced by HG, we conducted WB assays. WB indicated that HG promoted Pin1, BRD4, and NAP1L1 protein expression while inhibiting P21 expression in GC cells in a time-dependent manner (Fig. 4a and S6). When *Pin1* was silenced and HG medium was added, WB and qRT-PCR results showed that the expression levels of Pin1, BRD4, and NAP1L1 in GC cells were significantly inhibited, while P21 expression was significantly increased (Fig. 4b, c and S7). When *BRD4* was inhibited with JQ1 and HG medium was added, WB results showed that it significantly repressed BRD4 and NAP1L1 expression levels and increased P21 expression, although BRD4 showed no repressive effect on Pin1 expression (Fig. 4d and S8). These experiments established that the *Pin1/BRD4* axis regulates *P21* by indirectly targeting *NAP1L1*.

### Hyperglycemia-induced *Pin1/BRD4* axis promoted GC formation in vivo

**Fig. 5:**
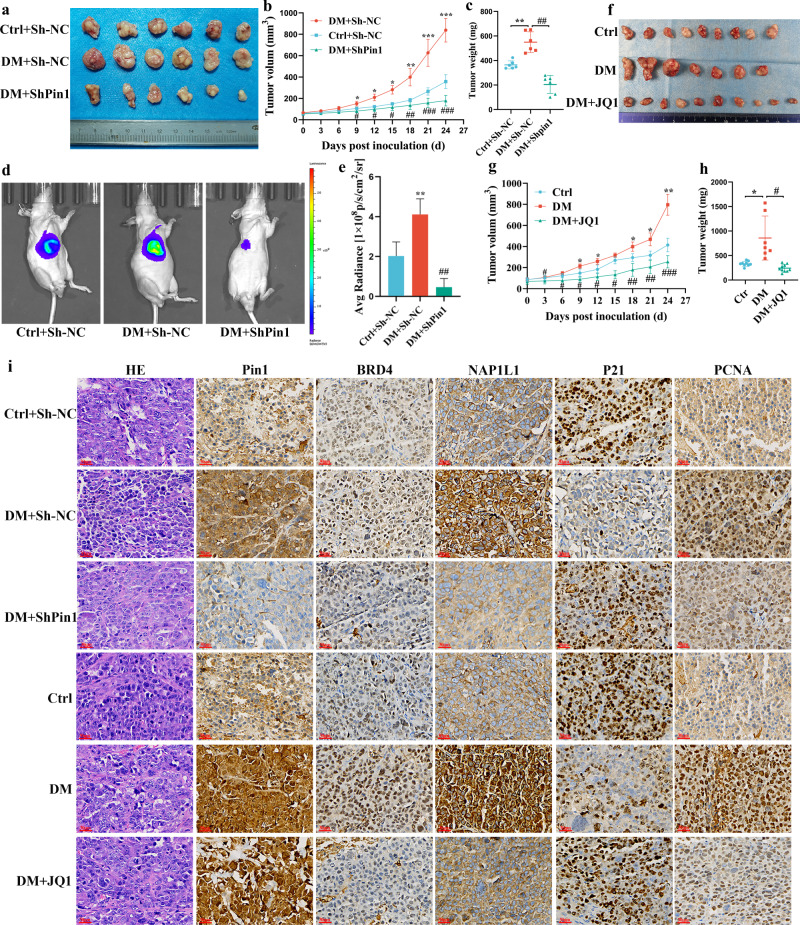
Hyperglycemia-induced *Pin1/BRD4* axis promoted GC formation in vivo. **a** Stable ShPin1 or Sh-NC MKN45 cells were injected subcutaneously into the right flank of nude mice. Tumor tissues were collected and photographed to evaluate tumor xenograft size 24 d after injection. **b** Tumor growth was observed by measuring tumor volumes. Values are mean ± SEM (*n* = 6); ^*^P < 0.05 vs. Ctrl+Sh-NC; ^**^P < 0.01 vs. Ctrl+Sh-NC; ^***^P < 0.001 vs. Ctrl+Sh-NC; ^*#*^*P* < 0.05 vs. DM + Sh-NC; ^*##*^*P* < 0.01 vs. DM + Sh-NC; ^*###*^*P* < 0.01 vs. DM + Sh-NC. **c** Tumor masses were weighed at indicated time points. ^**^P < 0.01 vs. Ctrl+Sh-NC; ^*##*^*P* < 0.01 vs. DM + Sh-NC. **d** In vivo growth of tumor xenografts was assessed using bioluminescence imaging. **e** Average radiance (p/s/cm^2^/sr) of tumor xenografts in the 3 groups at 24 d. Values are mean ± SEM (n = 6); ^**^P < 0.01 vs. Ctrl+Sh-NC; ^*##*^*P* < 0.01 vs. DM + Sh-NC. **f** MKN45 cells were injected subcutaneously into the right flank of nude mice and the DM + JQ1-group mice (n = 10) were treated with JQ1 (50 mg/kg) via intraperitoneal infusion. Tumor tissues of mice were collected and photographed to evaluate tumor xenograft sizes 24 d after injection. **g** Tumor growth was observed by measuring tumor volumes every three days with calipers. Values are mean ± SEM; ^*^P < 0.05 vs. Ctrl (*n* = 9); ^**^P < 0.01 vs. Ctrl; ^*#*^*P* < 0.05 vs. DM (*n* = 7); ^*##*^*P* < 0.01 vs. DM; ^*###*^*P* < 0.01 vs. DM. **h** Tumors were weighed at indicated time points. ^*^P < 0.05 vs. Ctrl; ^*#*^*P* < 0.05 vs. DM. **i** Immunohistochemistry of tumor tissues was used to detect Pin1, BRD4, NAP1L1, P21, and PCNA expression levels. The first picture on the left represents HE-stained tumors in each group. Scale bars, 30 μm (3 independent experiments performed).

To investigate regulation of the tumorigenesis function of the *Pin1/BRD4* axis by hyperglycemia in vivo, nude mouse hyperglycemic xenograft models were constructed by injection with STZ. We investigated the potential role of *Pin1* in tumorigenesis by subcutaneously injecting MKN45 cells stably transfected with shPin1 or sh-NC into hyperglycemic and normoglycemic nude mice, respectively. The DM + Sh-NC group grew larger and faster tumors, and displayed larger weights and volumes, compared with the Ctrl+Sh-NC group. Suppression of *Pin1* significantly inhibited tumor weight and volume in hyperglycemic mice (Fig. 5a–c). Similar results were observed for tumor fluorescence intensity of the mice via fluorescence imaging in vivo (Fig. 5d, e). Next, to investigate the potential role of *BRD4* in tumorigenesis, MKN45 cells were subcutaneously injected into hyperglycemic and normoglycemic nude mice. Inhibition of *BRD4* through JQ1 treatment in the DM + JQ1 group significantly inhibited tumor growth induced by hyperglycemia in hyperglycemic mice (Fig. 5f–h). The underlying mechanisms were further confirmed via HE and IHC staining. Compared with the Ctrl or Ctrl+Sh-NC group, tumor tissues derived from the HG or HG + Sh-NC group exhibited increased positivity for Pin1, BRD4, NAP1L1, PCNA, and MMP9, and reduced positivity for P21. Compared with the DM or DM + Sh-NC group, the DM + ShPin1 or DM + JQ1-group tumor tissues exhibited reduced positivity for BRD4, NAP1L1, PCNA, and MMP9, and increased positivity for P21, while JQ1 treatment had no effect on Pin1 expression (Fig. 5i).

### Effect of the *Pin1/BRD4* axis on lung metastasis of GC induced by hyperglycemia

**Fig. 6:**
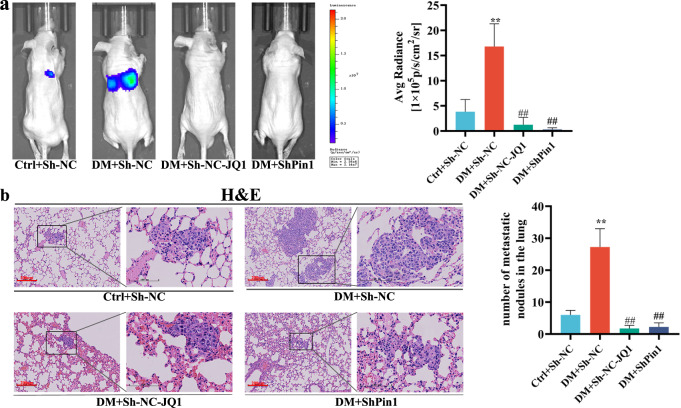
Effect of *Pin1/BRD4* axis on lung metastasis of GC induced by hyperglycemia. **a** Stable ShPin1 or Sh-NC MKN45 cells were injected into the tail vein of nude mice. In vivo tumor lung metastasis in tumor xenografts were assessed using bioluminescence imaging 24 days after injection. Lung metastases of the nude mice were quantified and measured as average radiance (p/s/cm^2^/sr). Values are mean ± SEM (n = 4); ^**^P < 0.01 vs. Ctrl+Sh-NC; ^*##*^*P* < 0.01 vs. DM + Sh-NC. **b** Lung tissues were stained with HE to observe lung metastasis nodules. Scale bars, 100 μm and 200 μm. The histogram shows the number of lung metastasis nodules. Values are mean ± SEM (n = 4). ^**^P < 0.01 vs. Ctrl+Sh-NC; ^*##*^*P* < 0.01 vs. DM + Sh-NC.

To further verify the effect of *Pin1/BRD4* axis on lung metastasis of GC induced by hyperglycemia in vivo, luciferase-labeled MKN45 (stably transfected with the sh-NC or shPin1 vector) was introduced into hyperglycemic and normoglycemic nude mice via tail-vein injection, following which DM + JQ1-group mice were treated with JQ1. In mouse lung metastasis nude models, the fluorescence intensity and the numbers of metastatic tumor nodules in the lungs of the DM + Sh-NC group were significantly increased compared with those of the Ctrl+Sh-NC group. Inhibition of *Pin1* or *BRD4* significantly inhibited pulmonary metastasis induced by hyperglycemia (Fig. 6a). HE staining substantiated these results (Fig. 6b). In summary, hyperglycemia-induced *Pin1/BRD4* axis promoted gastric tumorigenicity and tumor metastasis by facilitating *NAP1L1* and repressing *P21* in vivo.

## Discussion

Together, cancer and diabetes mellitus cause high mortality rates worldwide [25]. GC is an aggressive and lethal digestive cancer [26]. The known risk factors for GC include hyperglycemia, hyperlipidemia, and HP infection, although the underlying mechanisms remain unclear [27–29]. Several studies have identified hyperglycemia as being associated with various cancers [30]. The progression of various cancers becomes more aggressive with hyperglycemia [31]. Our previous Fujian prospective investigation of cancer (FIESTA) study also supports this notion, having showed that metabolic syndrome, especially hyperglycemia, is associated with increased risk for GC-related mortality and that hypoglycemic therapy significantly prolongs the survival time of patients with GC complicated by diabetes [32]. Given that changes in human lifestyle have led to increased metabolic disorders and abnormal glucose metabolism in cancer cells, the promotion of cancer development by hyperglycemia deserves intense investigation.

Hyperglycemia and obesity promote cancer cell proliferation by altering oncogenes and glucose metabolism at molecular levels [33]. Hyperglycemia independently or jointly promotes tumor progression within the tumor microenvironment, by activating the abnormally upregulated expression of epidermal growth factor and its receptor *EGFR* in cancer. Activation of EGFR stimulates protein kinase C and peroxisome proliferator-activated receptor-γ expression, and alters the *leptin/IGFR1* or *AKT/mTOR* signaling pathways [19, 34–36]. However, whether HG promotes proliferation and migration of GC cells via the *Pin1/BRD4* axis warrants further investigation.

Elevated Pin1 expression promotes cancer progression by disrupting the balance between oncogenes and tumor suppressors. *Pin1* regulates proliferation and migration of cancer by modulating P21 transactivation [37]. EGF-induced nuclear translocation of *PKM2* is regulated by *Pin1* to induce the Warburg effect and regulate glycolytic gene expression [38]. The Warburg effect increases aerobic glycolysis, enabling tumor development and suggesting that cancers are metabolic diseases [39]. Considering that hyperglycemia is an inherent biological feature of diabetes, and glucose an important energy source for cancer cells, it is apparent that hyperglycemia may play an important role in the progression of cancer in diabetic cancer patients. *Pin1* plays an important role in the glucose metabolism of tumor cells. Meanwhile, *BRD4* is regulated by *Pin1*. The present study aimed to explore the underlying mechanism in which *Pin1* and *BRD4* regulate the promotion of diabetes by GC.

*BRD4*, a functional component of cell-cycle control proteins, is a transcriptional factor and epigenetic regulator that plays a key role in embryonic development and carcinogenesis [40]. We previously elucidated that overexpressed BRD4 promotes GC cell proliferation by regulating P21 expression [19]. *NAP1L1*, which inhibits P21 expression via the *AKT* signal pathway, leading to abnormal proliferation of GC cells, is highly associated with GC cell proliferation [24]. Therefore, we speculate that *BRD4* regulates *P21* by targeting *NAP1L1*.

Here, we investigated whether the HG-microenvironment-induced *Pin1/BRD4* axis promotes proliferation, migration, and G1/S transition of GC cells, by suppressing P21 expression via targeting NAP1L1. After the expression of Pin1 or BRD4 was silenced, the expression levels of cyclin D1, MMP9, PCNA, and BCL-2 decreased, while Bax expression increased. These results demonstrated that regulation of Pin1 and BRD4 expression levels affected the proliferation and migration of GC cells in a HG microenvironment. We found that silencing Pin1 expression inhibited BRD4, and that inducing BRD4 expression had no significant effect on Pin1 expression. This is consistent with a study by Hu et al. [20].

This study had some limitations. First, we explored the effect of HG on GC and its underlying mechanism using only animal and cell studies and did not conduct a clinical sample study. Thus, the results do not represent the clinical situation well; clinical studies are required to validate our hypothesis. Second, with regard to the mechanism of *Pin1/BRD4* axis function in GC induced by HG, the evidence is insufficient to prove the interaction between BRD4 and NAP1L1, or between NAP1L1 and P21. Therefore, the study needs to be improved by using techniques such as co-immunoprecipitation assays to detect protein–protein interactions.

In summary, we revealed the critical role of *Pin1/BRD4* axis in the association between hyperglycemia and GC. Through in vivo and in vitro studies, we found that hyperglycemia induces Pin1 to promote the expression of BRD4, which prompts NAP1L1 to downregulate P21, resulting in the dysregulation of GC cell cycle, which leads to aberrant GC cell proliferation and migration, thereby enhancing GC progression in diabetics. This study elucidated the role played by the *Pin1/BRD4* pathway in diabetes- induced GC progression, and provides a theoretical basis for finding and screening new targets for antidiabetic tumor drugs.

## Materials and methods

### Antibodies, reagents, and kits

Primary and secondary antibodies used in this study are shown in Supplemental Table 1. Polyvinylidene difluoride (PVDF) membranes were purchased from Millipore (Darmstadt, Germany). The drugs used were as follows: streptozotozin (STZ, S0130); RPMI 1640 medium without glucose (R1383); (+)-JQ1 (BRD4 inhibitor, SML1524); and D-glucose (G7021), all purchased from Sigma-Aldrich (St Louis, MO, USA); and D-Luciferin potassium salt (C3654) purchased from APExBIO (Houston, TX, USA).

### Lentiviral infection

Short-hairpin RNA (shRNA) lentiviral vectors needed to target human *Pin1* were designed, validated, and synthesized by Hanbio Biotechnology Co. Ltd. (Shanghai, China). GC cells carrying stably knocked-down *Pin1* were established via transfection with an shPin1 lentivirus, according to standard procedures. To select stably transfected cells, flow cytometry (FACS Aria III cell sorting system, BD Biosciences, CA, USA) analysis was used. Multiplicity-of-infection efficiency was measured by counting GFP-positive cells with a > 90% infection efficiency using Olympus CK2 inverted microscope (Olympus Corporation, Tokyo, Japan). Stably transfected cells were selected for further analysis. ShRNA sequences used in this study are shown in Supplemental Table 2.

### Cell culture

The GC cell lines AGS, HGC27, and MKN45, obtained from the Cell Bank of the Chinese Academy of Sciences (Shanghai, China), were cultured in RPMI 1640 medium with 10% fetal bovine serum (FBS, Gibco, MA, USA), 100 U/ml penicillin, and 100 g/ml streptomycin. The origin of GC cells was authenticated by STR analysis. All cells were routinely tested for mycoplasma and found to be mycoplasma free. GC cells were grown to 80–90% confluency, starved for 24 h, and grouped as follows: ctrl (D-glucose: 5.5 mM); mannitol (D-glucose: 5.5 mM + mannitol: 19.5 mM); HG (D-glucose: 25 mM); HG + JQ1 (JQ1: 10–6 M); HG + ShPin1; and HG + Sh-NC.

### Cell proliferation assay

For the Cell Counting Kit-8 assay (CCK-8), GC cells were seeded into 96-well tissue culture plates (Corning, NY, USA) at a density of 2000–5000 cells per well. Approximately 10 μl of CCK-8 kit (Beyotime Biotechnology, Jiangsu, China) was added to each well and incubated at 37 °C for 1 h. Cell growth was analyzed using a microplate reader (Spectra Max i3X, Molecular Devices, CA, USA) at 450 nm at 24, 48, 72, and 96 h. For the EdU incorporation assay, 10 μM EdU Reagent (Beyotime Biotechnology) was added to the cells and incubated at 37 °C for 2 h. A Cell-Light EdU DNA Cell Proliferation Kit (Beyotime Biotechnology) was used for Edu staining. Images were gathered via Olympus BX43 microscope (Olympus Company). Captured images were processed using Image Pro Plus 6.0 software (Media Cybernetics, USA) and Adobe Photoshop 2020 (Adobe Photoshop Inc., CA, USA). The proliferative ratio of GC cells was calculated by plotting the EdU-positive cells against total cells within each field.

### Cell migration assay

GC cell migration was examined via wound-healing and Transwell assays. Transwell assays were performed in Transwell inserts with an 8.0-μm Transwell Permeable Support chamber (Millipore). GC cells grown to 80–90% confluency and harvested for 24 h were seeded in the upper chamber (3 × 10^5^ cells per well), and culture medium (0.75 ml) containing 10% FBS was added to the lower chamber. The culture plate was incubated at 37 °C for 24 h, and cells in the lower-chamber membrane were fixed with 4% paraformaldehyde for 15 min and stained for 20 min with 0.1% crystal violet solution. Images were acquired using an inverted microscope. The number of GC cells was calculated using ImageJ software. Wound-healing scratch assays were performed according to standard protocol [41].

### Flow-cytometric analysis

Cell cycle was analyzed using a Cell Cycle Assay Kit (Beyotime Biotechnology). In brief, cells were collected and permeabilized in cold 70% ethanol overnight at 4 °C. Fixed cells were washed in PBS and stained with a solution containing propidium iodide (PI, 25 μg/ml) and RNAse (1 μg/ml) for 30 min at 37 °C in the dark. Cell-cycle distribution of cells was evaluated via flow cytometry in FACS Accuri C6 (BD BioSciences) and analyzed using FlowJo (version 10) software (FLOWJO LLC, Ashland, OR).

### Western blotting (WB)

SDS-PAGE and WB were performed according to standard methods. Protein bands were visualized via the FluorChem system (ProteinSimple, San Jose, CA, USA) using the ECL reagent (Beyotime Biotechnology).

### Quantitative real-time PCR (qRT-PCR)

qRT-PCR was performed as described in Supplemental Methods. Gene-specific primer sequences, purchased from Sangon Biotech (Shanghai, China), are listed in Supplemental Table 3.

### Tumorigenicity and metastasis assays in nude mice

All animal experiments were conducted in accordance with the National Institutes of Health Guide for the Care and Use of Laboratory Animals and approved by The Animal Ethics Committee of Fujian Medical University (research license number: FJMU IACUC 2020-0117). Male four-week-old athymic nude mice (BALB/c-nude), provided by GemPharmatech Co. Ltd. (Nanjing, China, Permit number: SCXK-2016-0010), were fed under specific pathogen-free (SPF) conditions. The mice were randomly separated into diabetic (DM) and nondiabetic (Ctrl) groups. Diabetic-group mice were intraperitoneally injected with STZ (100 mg/kg) diluted in cold sodium citrate buffer (pH: 4.2–4.5). Mice with fasting plasma glucose levels ≥14 mmol/L were considered to be diabetes mellitus (DM) models. Normal MKN45 cells or MKN45 cells with stably transfected shPin1 or sh-NC were suspended in 0.1 mL PBS (1 × 10^6^ cells) and injected into the right armpits or tail veins of BALB/c-nude mice. Tumor volumes were calculated using Vernier calipers every three days using the following formula: volume = [(short diameter)^2^ × (long diameter)/2]. The DM + JQ1-group mice were treated with JQ1 (50 mg/kg) once every two days. Tumor growth and metastasis were analyzed by assessing bioluminescent flux (photons/s/sr/cm^2^) using an IVIS spectrum imaging system (PerkinElmer, MA, USA) and Living Image software (PerkinElmer). On the 24th day after the tumor cell injection, tumors and lung tissues were collected for further analysis. Data were excluded from the analysis if an animal died during experiments.

### Hematoxylin and eosin (H&E) staining and immunohistochemistry (IHC) analysis

H&E staining and IHC analysis were performed according to standard protocols [13, 42]. Lung metastasis of GC was observed using H&E staining images and counted by analyzing the number of metastatic nodules. Primary antibodies for IHC analysis are shown in Supplemental Table 1. Semiquantitative analysis (H-score method) of IHC was performed as described in Supplemental Methods [13].

### Statistical analysis

Statistical methods for each figure are given in the Supplementary Methods.

## Supplementary information

Supplementary information

Supplementary information of WB

Author Contribution Statement

The ARRIVE guidelines

## Data Availability

The data supporting the findings of this work are available within the article and its Supplementary Information files. Partial source data are available in figshare (10.6084/m9.figshare.19556425). All data are available from the authors upon reasonable request.
