# Supplementary material for: Hyperglycemia induces gastric carcinoma proliferation and migration via the Pin1/BRD4 pathway
Source: Cell Death Discov. 2022 Apr 23;8:224. doi: 10.1038/s41420-022-01030-4 (PMC9035156; doi:10.1038/s41420-022-01030-4)
Supplement: Supplementary file 1 — Supplementary information [file 41420_2022_1030_MOESM1_ESM.pdf]

Supplementary information

Supplemental Methods

Quantitative real-time-PCR (qRT-PCR)

Total RNA was extracted from cultured cells using TRIzol reagent (Invitrogen, Carlsbad, USA) and reverse transcribed into complementary DNA using a Reverse Transcription kit (TaKaRa, Japan). Quantitative RT-PCR assay was performed using SYBR from Premix Ex Taq (Takara) in a StepOne Plus (Applied Biosystems, CA, USA), and human β-actin was used as internal control for mRNA detection. Gene expression was quantified using Ct values and normalized by the housekeeping gene β-actin via the 2-ΔΔCt method. Gene-specific primer sequences, purchased from Sangon Biotech (Shanghai, China), are listed in Supplemental Table 3.

Immunohistochemical assessment

Semi-quantitative analysis (H-score method) was performed according to Shenton's method with slight modifications. In brief, the percentages of positive aromatase-staining cells in tumor and stromal sections were subjectively assessed on five random visual fields for each specimen by two independent investigators (ZZ and HI) and discordant slides were solved by consultation with a third investigator, HY. The intensity of cytoplasmic staining was scored as 0, 1, 2, or 3, corresponding to negative, weak, intermediate, or strong immunoreactivity, respectively. The H-score (H) was calculated as follows: H = (% of cells that stained at intensity category 1 × 1) + (% of cells that stainedat intensity category 2 × 2) + (% of cells that stained at

intensity category 3 × 3).

Additionally, the immunostaining of ER-α and PgR were evaluated following the method [18]. In brief, the proportion of positive staining throughout the entire slide was assessed as 0 (negative), 1 (<1%),2 (1–10%), 3 (10%–1/3), 4 (1/3–2/3), and 5 (more than 2/3), and the average staining intensity was lodged 0(negative), 1 (weak), 2 (moderate) or 3 (strong) under light microscopy. The IHC score of each slide (0 or 2–8) was obtained as the sum of the proportion and intensity. ER-α and PgR status by IHC were then assessed as negative (scores 0 and 2) or positive (scores3–9) .

Statistical analysis

Statistical analyses were performed using SPSS software (version 16, SPSS, Chicago, IL). Results are presented as mean ± SEM. Data were analyzed using Student's t-test and one-way factorial ANOVA followed by post hoc comparisons. Statistical significance was set at $P < 0.05$. All assays were independently replicated at least three times.

Supplemental Tables

Supplemental Table 1. Antibodies used in the current study.

Target (Art.No.)	Application	Dilution	Vendor
Pin1(ab192036)	IHC Staining	1:1000	Abcam (Cambridge, UK)
BRD4 (ab128874)	IHC Staining	1:500	Abcam
NAP1L1 (ab178687)	IHC Staining	1:500	Abcam
PCNA (#2586)	IHC Staining	1:1000	Cell Signaling Technologies (CST, Danvers, MA, USA)
P21 (A19094)	IHC Staining	1:500	Abclonal (Wuhan, China)
Cyclin D1 (ab16663)	Western	1:1000	Abcam
MMP9 (#13667)	Western	1:1000	CST
Bcl-2 (A19693)	Western	1:1000	Abclonal
Bax (A20227)	Western	1:1000	Abclonal
β-actin (AC038)	Western	1:5000	Abclonal
Pin1(ab192036)	Western	1:1000	Abcam
BRD4 (ab128874)	Western	1:500	Abcam
NAP1L1 (ab178687)	Western	1:500	Abcam
PCNA (#2586)	Western	1:1000	CST
P21 (A19094)	Western	1:500	Abclonal
Goat anti-mouse IgG-HRP	Western	1:5000	Santa Cruz Biotechnology (CA, USA)
Goat anti-rabbit IgG-HRP	Western	1:5000	Santa Cruz Biotechnology

Supplemental Table 2. ShRNA sequences used in this study.

Gene	Top strand (5′-3′)	Bottom strand (5′-3′)
ShPin1#1	GATCCGCCATTTGAAGACGCCTCGTTTTCAAGAGAA<br>ACGAGGCGTCTTCAAATGGCTTTTTTG	AATTCAAAAAAGCCATTTGAAGACGCCTCGTTTCTCT<br>TGAAAACGAGGCGTCTTCAAATGGCG
ShPin1#2	GATCCGCAGTGGTGGCAAAAACGGGCTTCAAGAGA<br>GCCCGTTTTTGCCACCACTGCTTTTTTG	AATTCAAAAAAGCAGTGGTGGCAAAAACGGGCTCTC<br>TTGAAGCCCGTTTTTGCCACCACTGCG
ShPin1#3	GATCCGCCAGAAGATCAAGTCGGGAGATTCAAGAG<br>ATCTCCCGACTTGATCTTCTGGTTTTTTG	AATTCAAAAAACCAGAAGATCAAGTCGGGAGATCTC<br>TTGAATCTCCCGACTTGATCTTCTGGCG
Sh-NC	GATCCGTTCTCCGAACGTGTCACGTAATTCAAGAGA<br>TTACGTGACACGTTCGGAGAATTTTTTC	AATTGAAAAAATTCTCCGAACGTGTCACGTAATCTCT<br>TGAATTACGTGACACGTTCGGAGAACG

Supplemental Table 3. Primers used with qRT-PCR.

Species	Gene	Forward primer (5′-3′)	Reverse primer (5′-3′)
human	*Pin1*	TTCAGCAGAGGTCAGATG	CGGAGGATGATGTGGATG
human	*BRD4*	GCCTGGAGATGACATAGT	CGATGCTTGAGTTGTGTT
human	*NAP1L1*	GATGATGTTGAAGAAGTAGAAGA	CAATGTATCCTGTTGGTGTT
human	*P21*	TGTCCGTCAGAACCCATGC	AAAGTCGAAGTTCCATCGCTC
human	*β-actin*	AGAGGGAAATCGTGCGTGAC	GAAGGAAGGCTGGAAGAGAG

Supplemental Table 4. Relationship between Pin1 and BRD4 expression and clinicopathological features in 136 patients with gastric cancer [%]

Characteristics	No.(N=142) (%)	Pin1 positive(%)	*p* value	BRD4 positive(%)	*p* value
Tissues			0.018		0.000
Adjacent carcinoma tissues	50	8		4	
Cancer tissues	136	46		59	
Age			0.714		0.316
＜50	26	8		9	
⩾50	110	38		50	
T size			0.454		0.338
T1+T2	38	11		14	
T3+T4	98	35		45	
N status			0.400		0.501
N0	41	16		16	
N+	95	30		43	
M status			0.979		0.239
M0	130	44		55	
M+	6	2		4	
TNM stage			0.983		0.247
I+II	56	19		21	
III+IV	80	27		38	
Differentiation			0.280		0.051
Low	77	29		39	
High	59	17		20	

Supplemental Figures

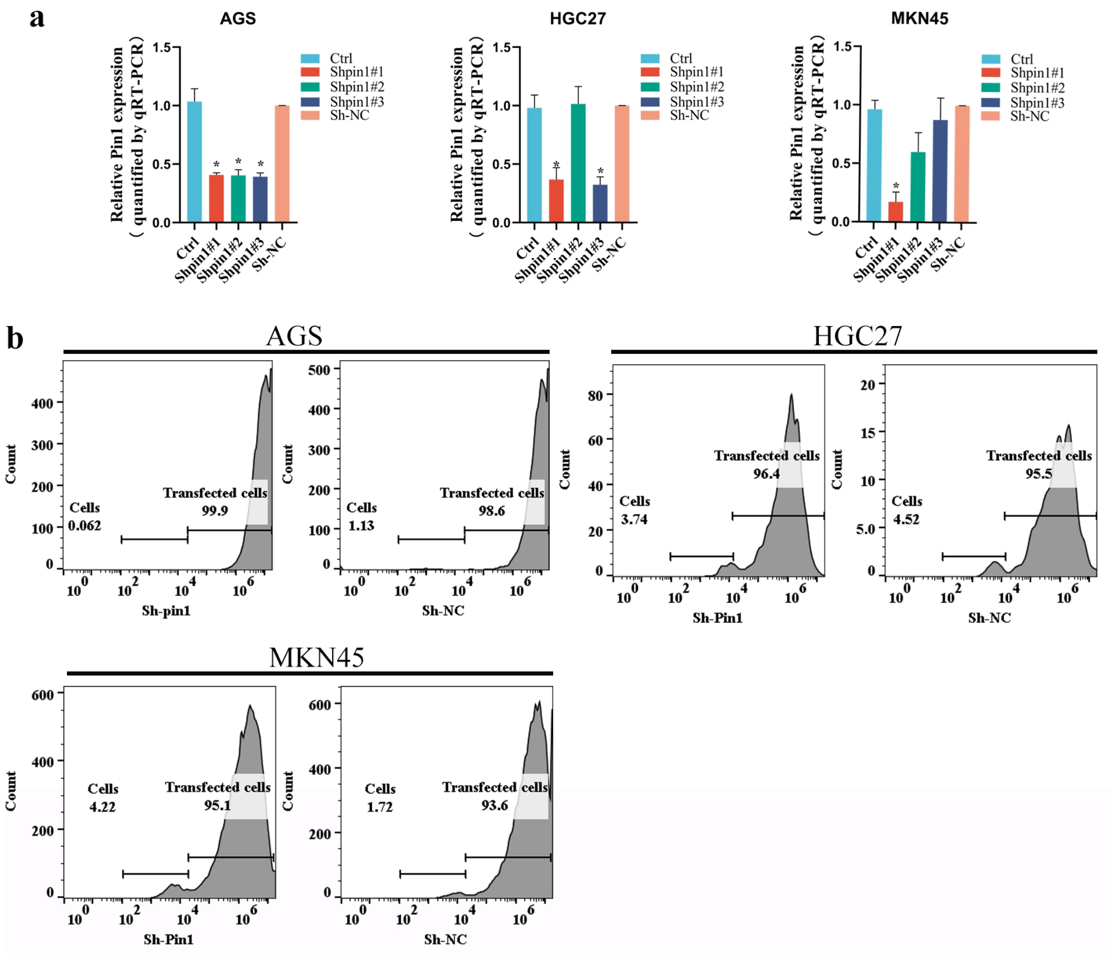

Fig. S1. Lentivirus vector was used to establish the stable *Pin1* knockdown gastric cancer cell lines. (a). Gastric cancer cells were transfected with lentivirus vector of ShPin1 or Sh-NC. Total RNA of gastric cancer cells were extracted and Pin1 mRNA expression levels were measured by qRT-PCR. (b). Flow-cytometric sorting was used to screen the transfected GFP-positive cells. Values are mean ± SEM (at least n=3 experiments). $^{*}P < 0.05$ vs. Ctrl.

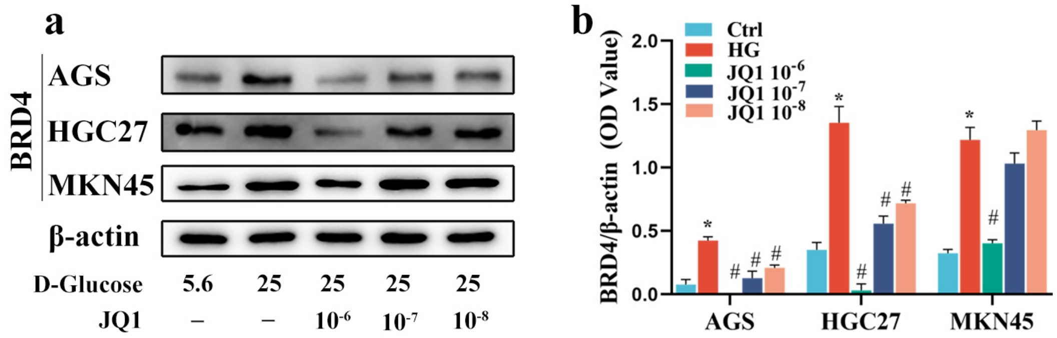

Fig. S2. JQ1 reduce upregulation of BRD4 protein expression induced by HG in a dose-dependent manner. (a). JQ1 inhibited high glucose-induced upregulation of BRD4 protein expression in a dose-dependent manner. Gastric cancer cells were pretreated with different concentrations of JQ1 (10^{-6} – 10^{-8} M) for 1 h and incubated with high glucose (25 mM) for 72 h. The protein were extracted from cells for western blotting. β-actin served as the loading control. (b). Values are mean ± SEM (at least n=3 experiments). $^{***}P < 0.001$ vs. Ctrl; $^{\#}P < 0.05$ vs. HG; $^{\#\#}P < 0.01$ vs. HG; $^{\#\#\#}P < 0.001$ vs. HG.

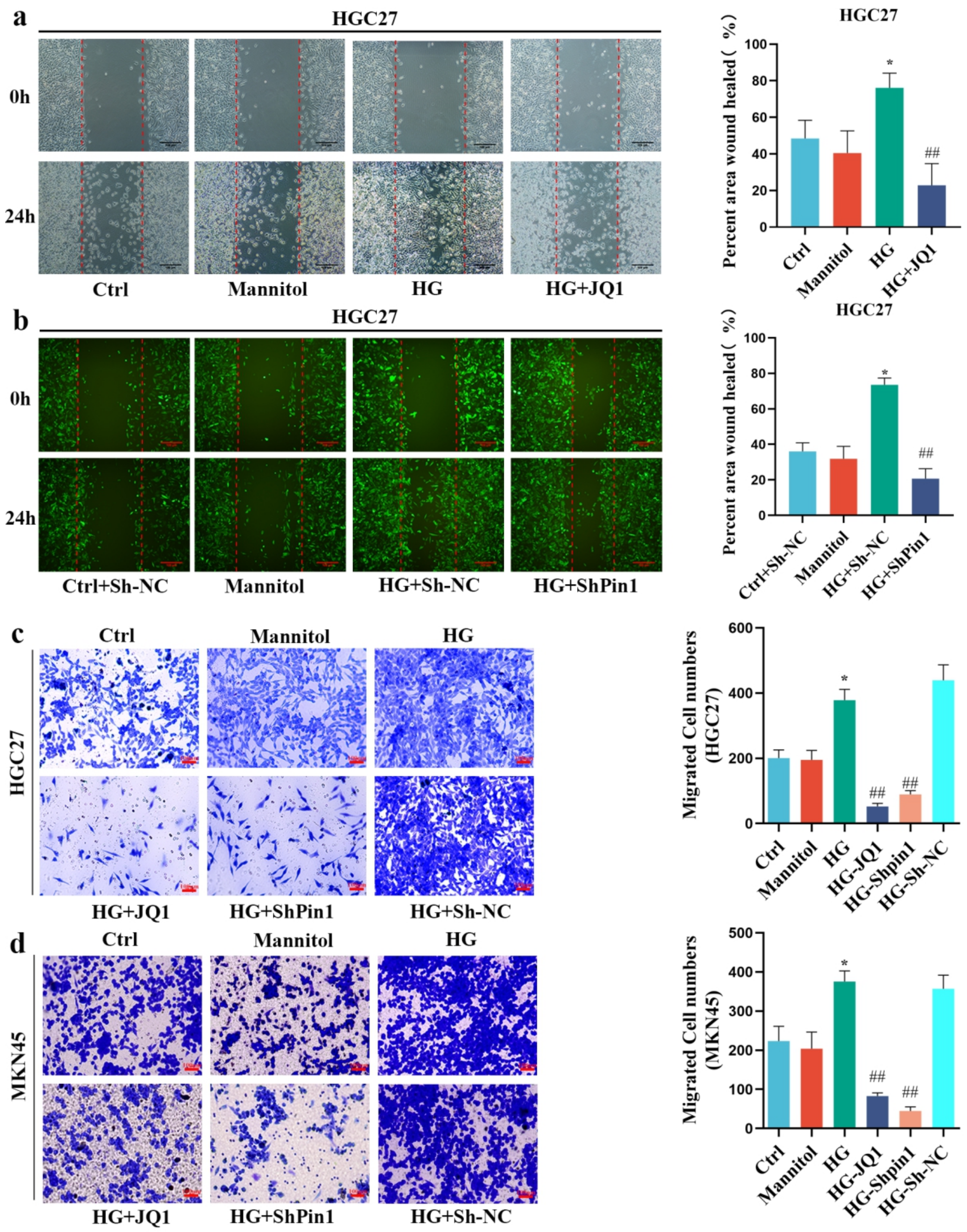

Fig. S3. Effect of silencing *Pin1 or BRD4* on migration of HGC27 and MKN45 cells induced by high glucose (a). Wound-healing assay were used to measure migration of HGC27. HGC27 cells were treated with JQ1 (10^{-6} M) and incubated with high glucose (25 mM) for 24 h. Values are mean ± SEM (at least n=3 experiments). $^{*}P < 0.05$ vs. Ctrl; $^{\#\#}P < 0.01$ vs. HG. (b). Wound healing assay of HGC27 transfected with the lentivirus vector of ShPin1 or Sh-NC. Values are mean ± SEM (at least n=3 experiments). $^{*}P < 0.05$ vs. Ctrl+Sh-NC; $^{\#\#}P < 0.01$ vs. HG+Sh-NC. (c, d). Tanswell assay were used to measure migration of HGC27 and MKN45 cells. Scale bars, 100 μm. Values are mean ± SEM (at least n=3 experiments). $^{*}P < 0.05$ vs. Ctrl; $^{\#\#}P < 0.01$ vs. HG.

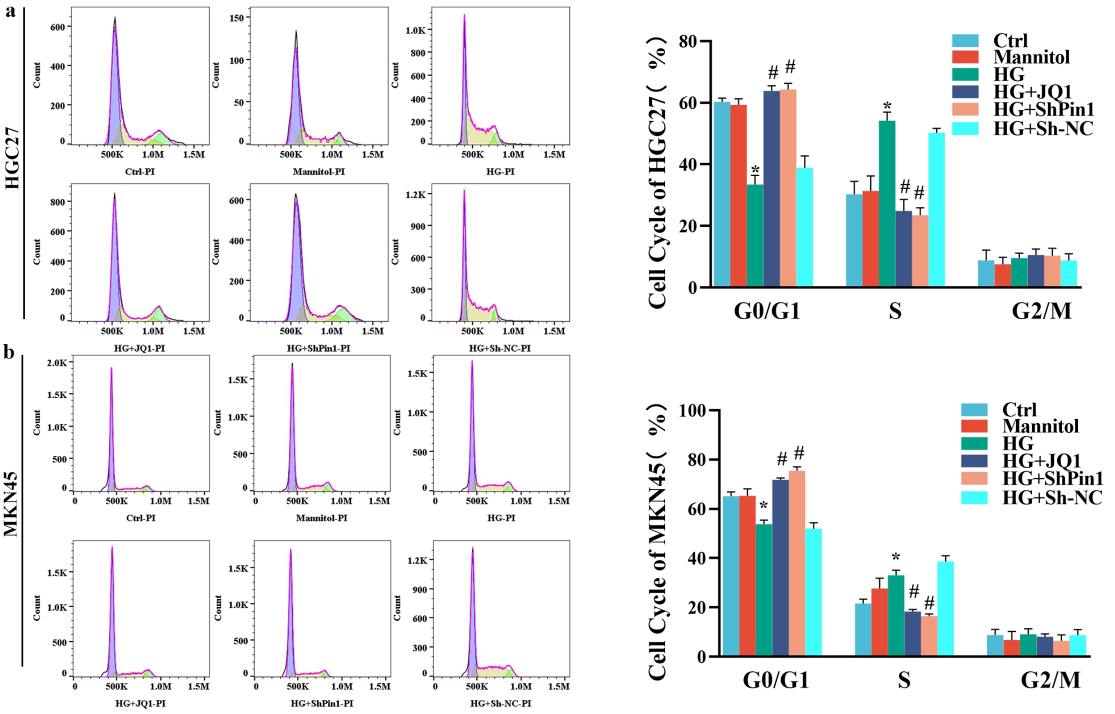

Fig. S4. Effect of down-regulation of *Pin1* or *BRD4* on High glucose-induced cell cycle. Gastric cancer cells were infected with lentivirus vector of ShPin1 or Sh-NC or were treated with JQ1 (10^{-6} M) and then incubated with high glucose (25 mM) for 72 h. After this time: (a, b). cells fixed, stained with propidium iodide and each group cell cycle profile determined by flow cytometry.

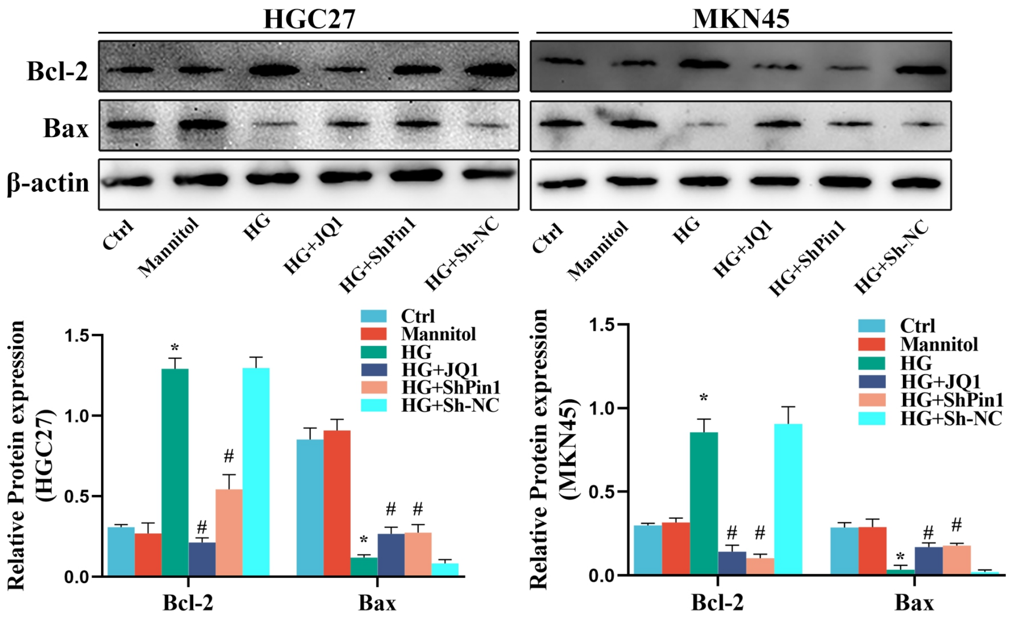

Fig. S5. Effect of silencing *Pin1* or *BRD4* gene on migration of HGC27 and MKN45 cells induced by high glucose HGC27 and MKN45 cells were infected with lentivirus vector of ShPin1 or Sh-NC or were treated with JQ1 (10-6 M) and then incubated with high glucose (25 mM) for 72 h. After this time, The protein of Bcl-2 and Bax were extracted from cells for western blotting, β-actin served as the loading control; Values are mean ± SEM (at least n=3 experiments). $^{*}P < 0.05$ vs. Ctrl; $^{\#}P < 0.05$ vs. HG.

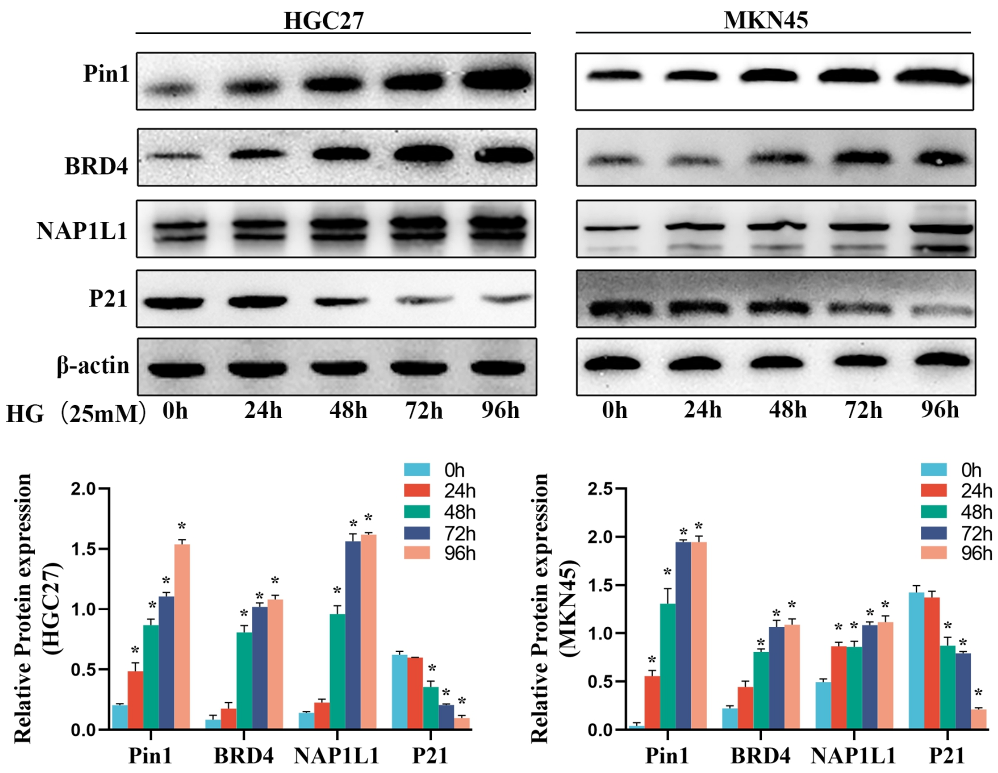

Fig. S6. High glucose regulates Pin1 protein expression of gastric cancer cells in a time-dependent manner. HGC27 and MKN45 were treated with high glucose (25 mM) for 24, 48, 72, and 96 h. Pin1, BRD4, NAP1L1 and P21 protein expression was detected by Western Blotting, β-actin served as the loading control; Values are mean ± SEM (at least n=3 experiments); $^{*}P < 0.05$ vs. 0h.

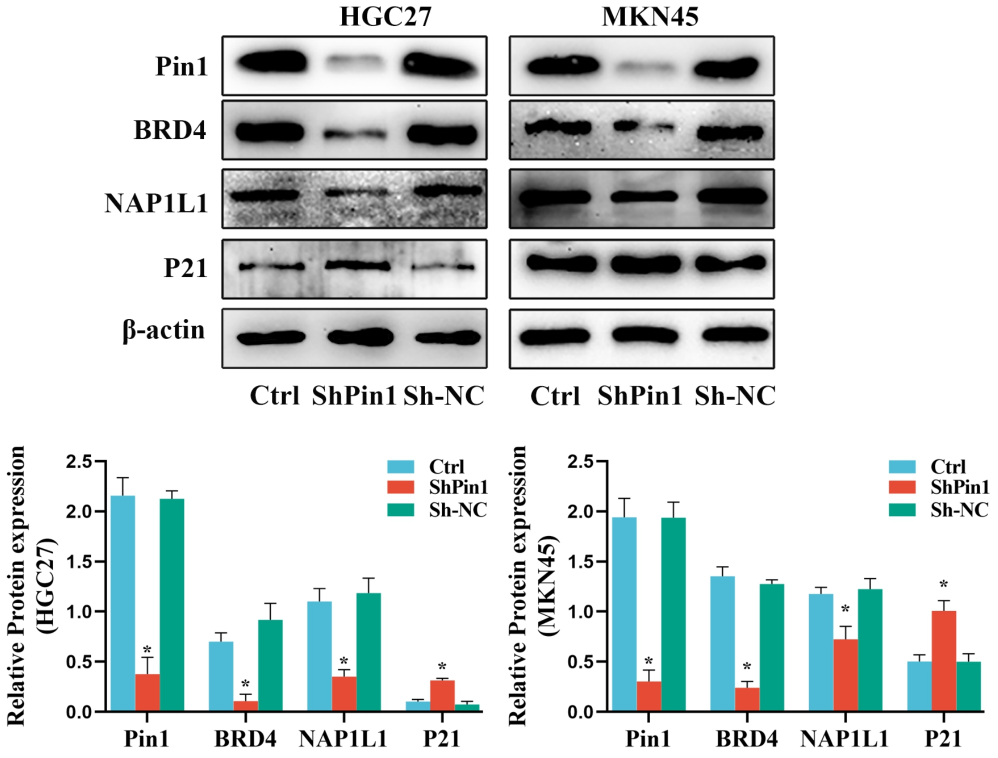

Fig. S7. Silencing *Pin1* were significantly inhibited the expression of BRD4 and NAP1L1 protein and promote the expression of P21 in gastric cancer cells. HGC27 and MKN45 cells were infected with lentivirus vector of ShPin1 or Sh-NC and then incubated with high glucose (25 mM) for 72 h. After this time, The protein of Pin1, BRD4, NAP1L1 and P21 were extracted from cells for western blotting, β-actin served as the loading control; Values are mean ± SEM (at least n=3 experiments). $^{*}P < 0.05$ vs. Ctrl;

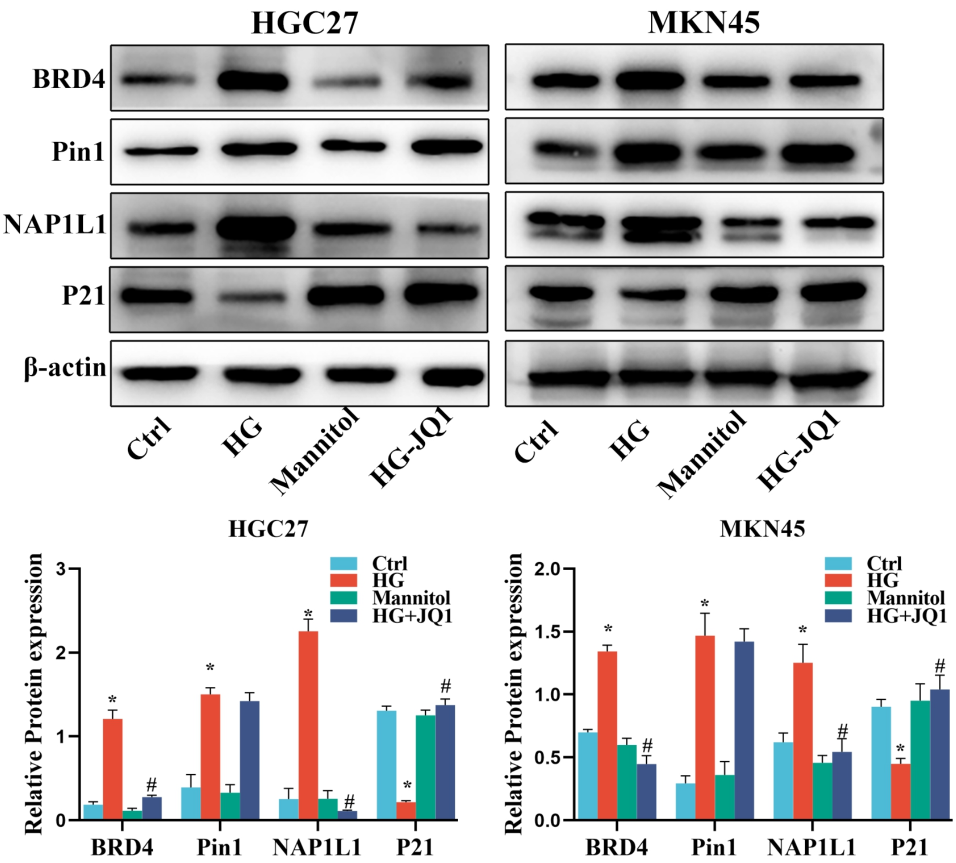

Fig. S8. Inhibition of *BRD4* could significantly inhibit the expression of NAP1L1 protein and promote the expression of P21 in gastric cancer cells induced by high glucose, but had no significant effect on Pin1 protein expression. HGC27 and MKN45 cells were were pretreated with JQ1 (10^{-6} M) for 1 h and then incubated with high glucose (25 mM) for 72 h; Pin1, BRD4, NAP1L1 and P21 protein were detected by Western Blotting, β-actin served as the loading control; Values are mean ± SEM (at least n=3 experiments); $^{*}P < 0.05$ vs. Ctrl; $^{\#}P < 0.05$ vs. HG.

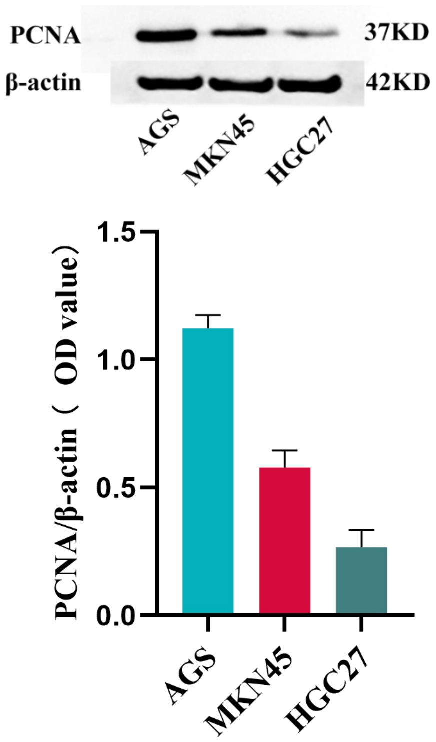

Fig. S9. The expression of PCNA protein in AGS, HGC27 and MKN45 cells PCNA protein were detected by Western Blotting, β-actin served as the loading control; Values are mean ± SEM (at least n=3 experiments);

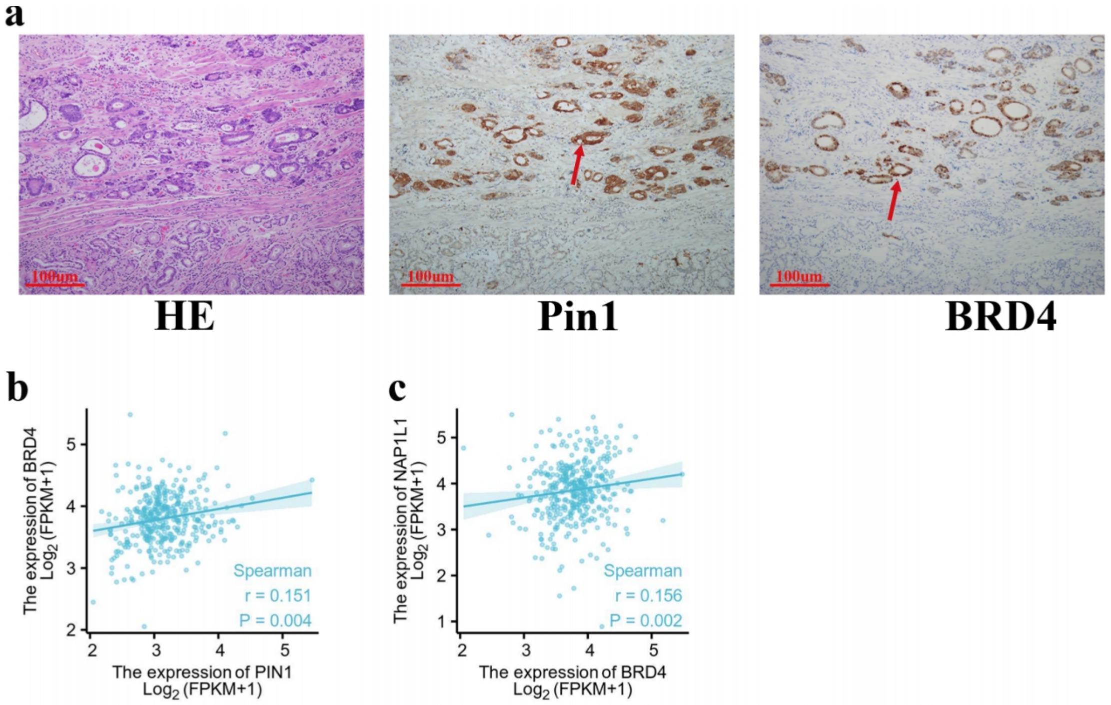

Fig.S10 (a). Differential expression of Pin1 and BRD4 in gastric cancer and para-cancerous tissues. Arrowhead: cancer tissues.Scale bars, 100 μm. (b). The relationship between Pin1 and BRD4 predicted by bioinformatic analysis. The data comes from TCGA database (https://portal.gdc.cancer.gov/).(c). The relationship between BRD4 and NAP1L1 predicted by bioinformatic analysis. The data comes from TCGA database (https://portal.gdc.cancer.gov/) .

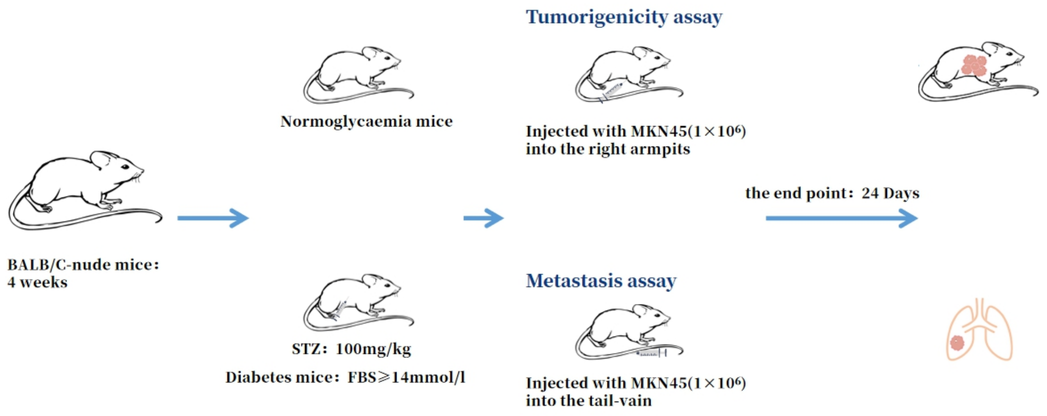

Fig.S11 The flow chart of the animal experiment
