# Supplementary material for: Hyperglycemia induces gastric carcinoma proliferation and migration via the Pin1/BRD4 pathway
Source: Cell Death Discov. 2022 Apr 23;8:224. doi: 10.1038/s41420-022-01030-4 (PMC9035156; doi:10.1038/s41420-022-01030-4)

Fig 1 WB Original Data_PCNA_Actin

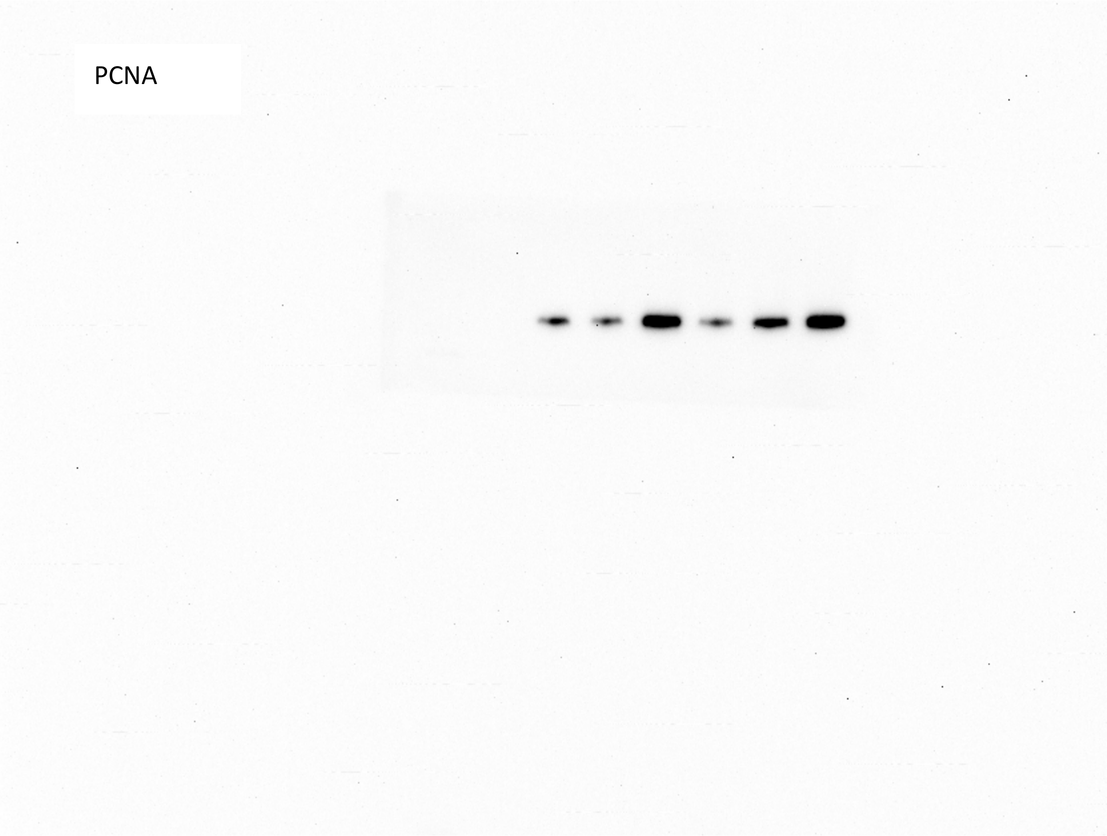

PCNA

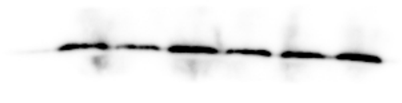

PCNA

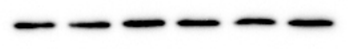

Actin

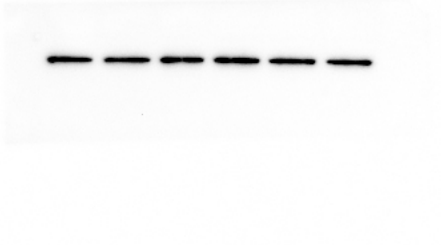

Fig 2 WB Original Data_MMP9_actin

MMP9

100
70

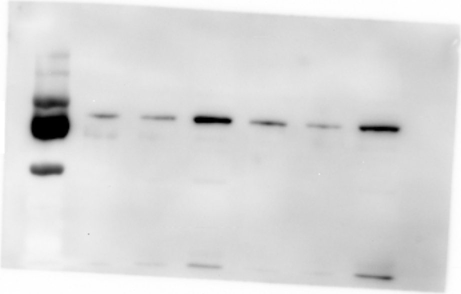

MMP9

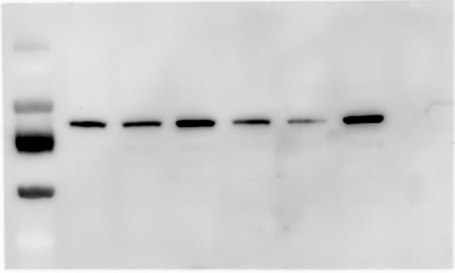

MMP9

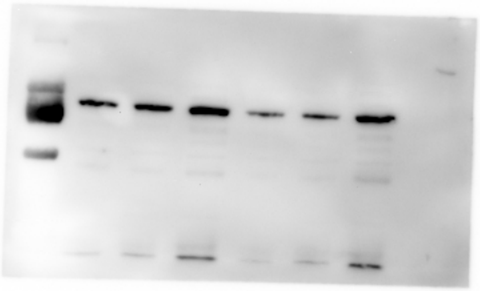

Actin

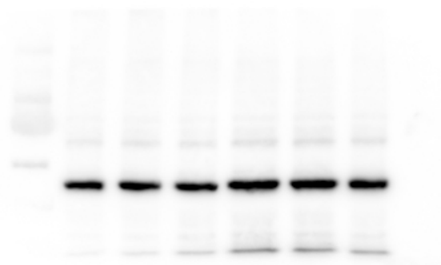

55

Fig3 WB Original Data_Cyclin D1_Actin

Cyclin D1

55
40

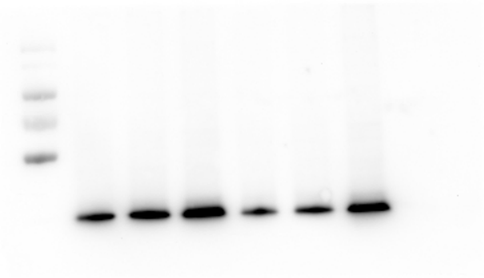

CyclinD1

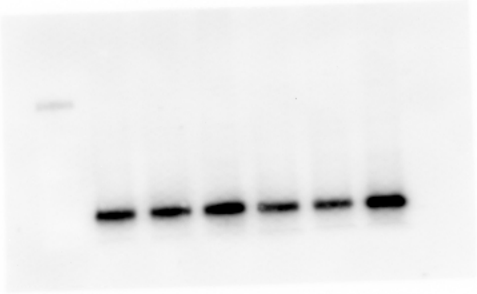

Cyclin D1

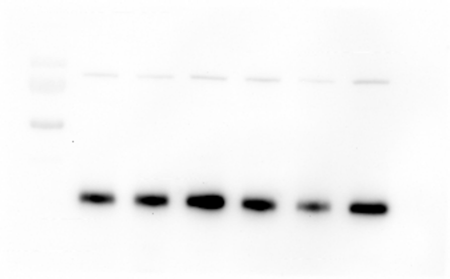

Actin

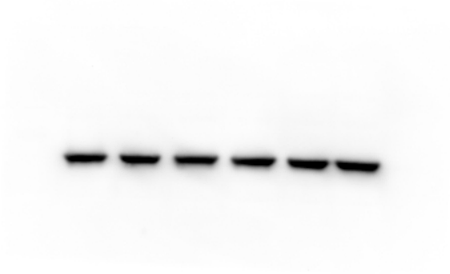

Fig3 WB Original Data_Bcl2_Bax_Actin

Bcl-2

40
35

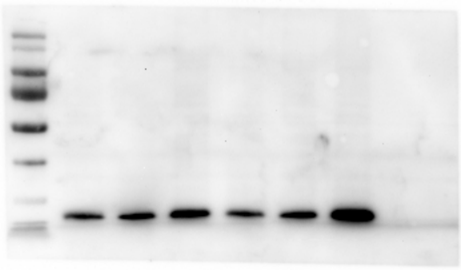

Bax

70

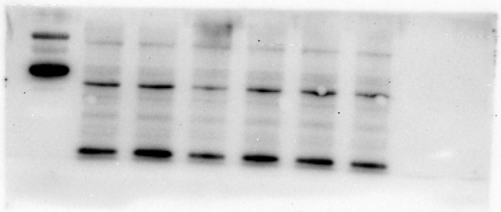

Actin

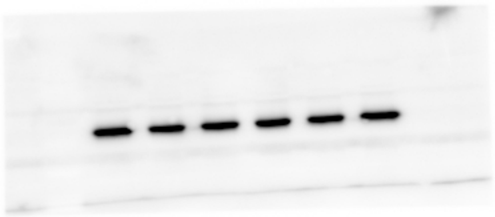

Fig4.(a). WB Original Data_Pin1_BRD4_NAP1L1_P21_Actin

Pin1

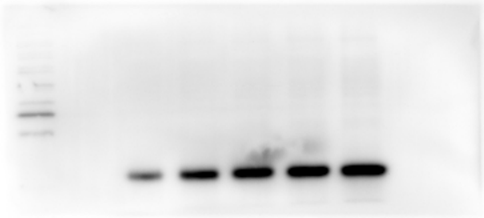

18KD

BRD4

180

130

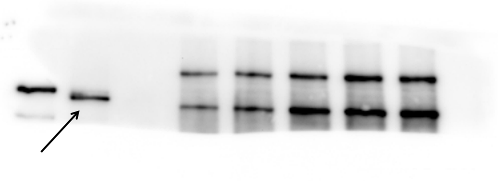

160KD

Nap1l1

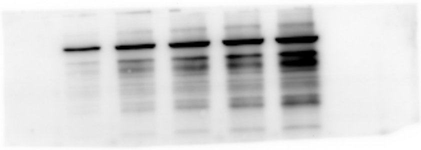

P21

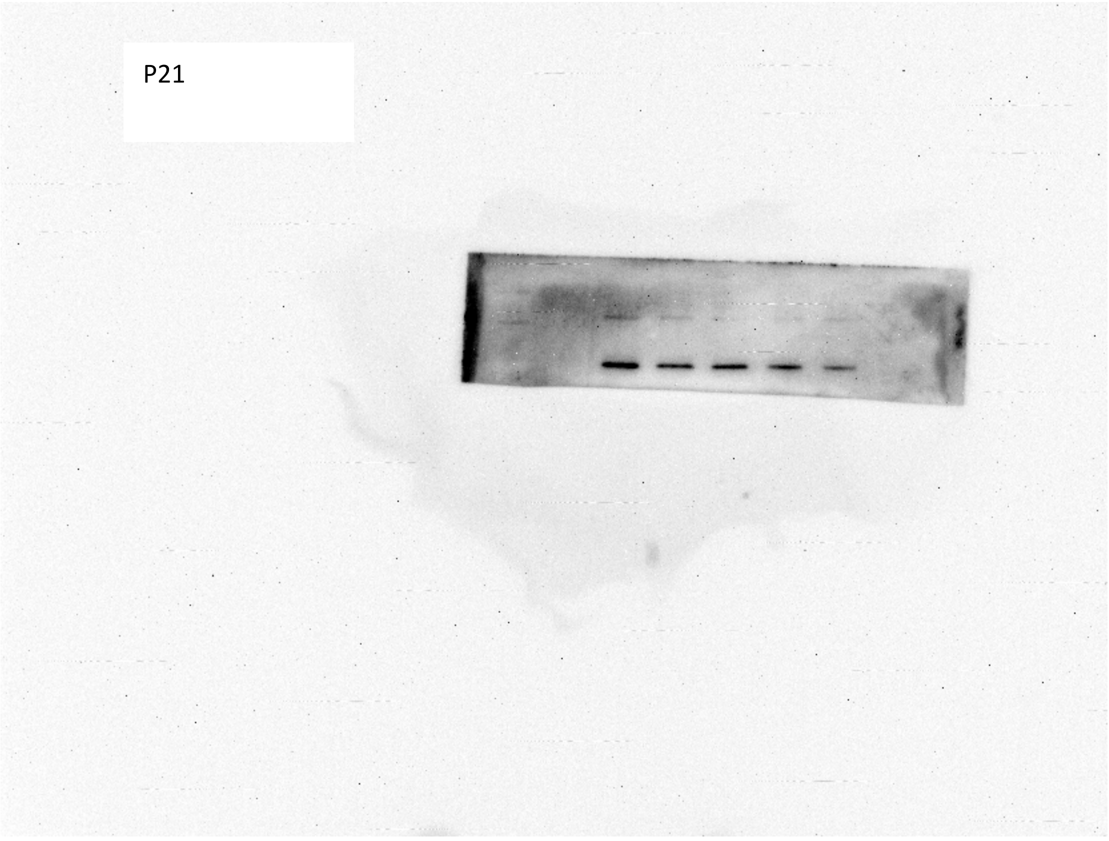

Actin

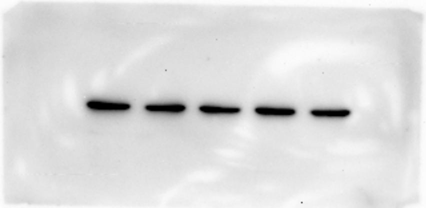

Fig4.(b). WB Original Data_Pin1_BRD4_NAP1L1_P21_Actin

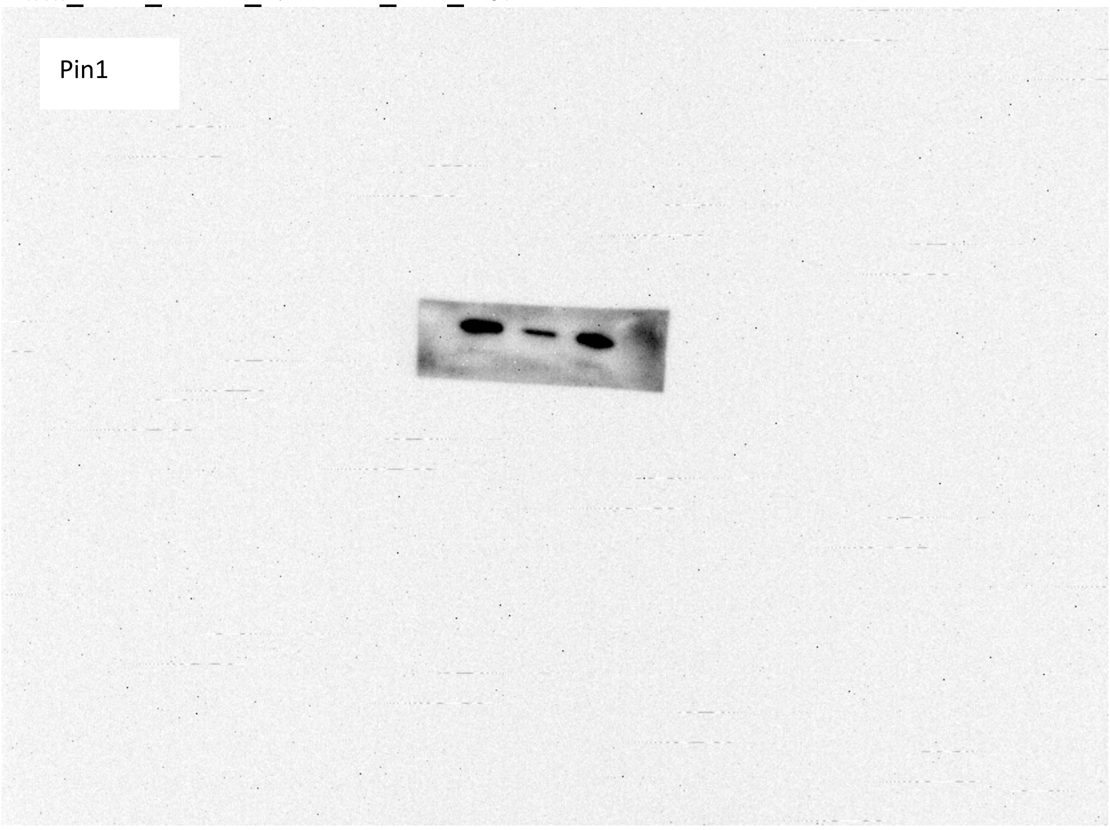

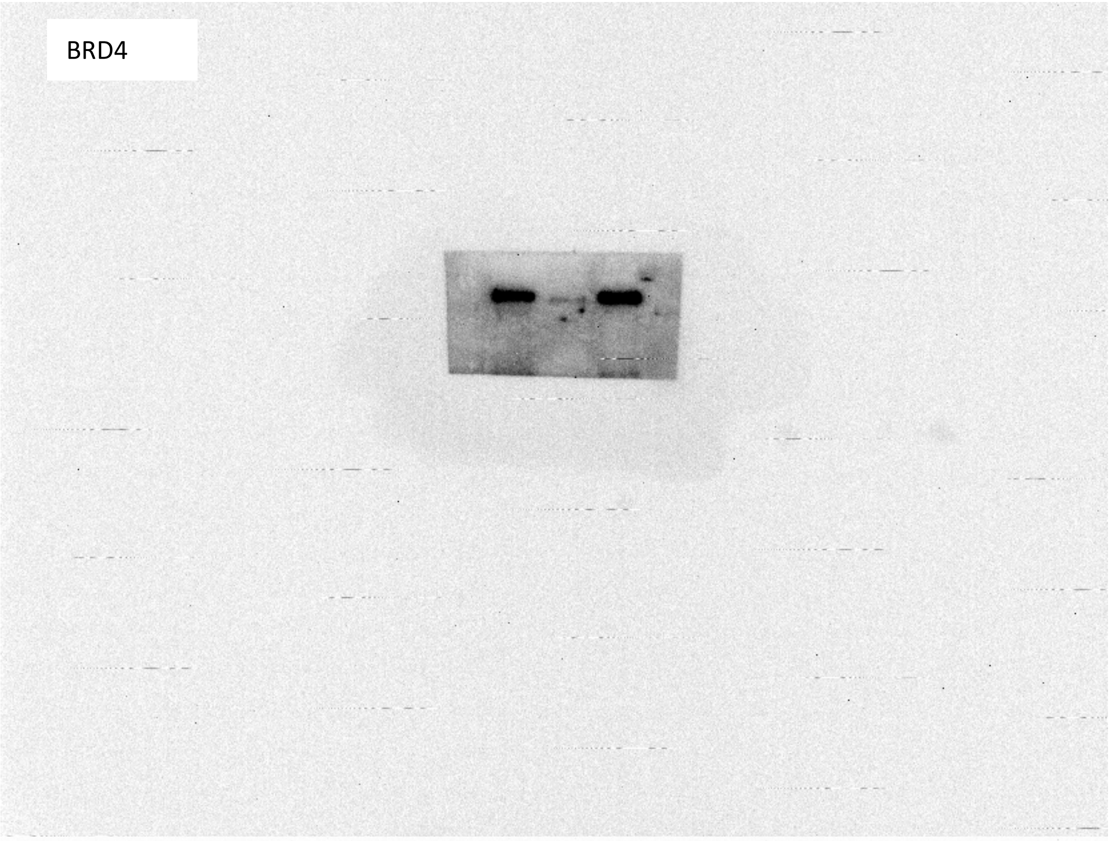
BRD4

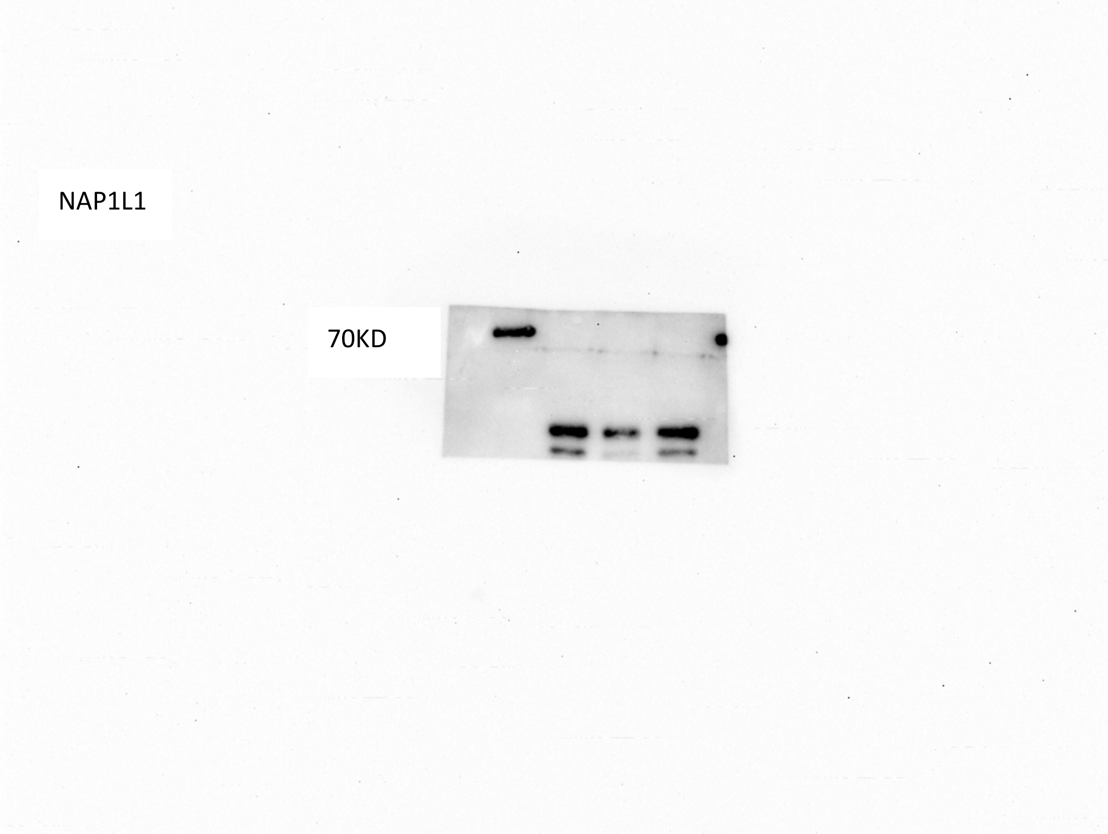
NAP1L1
70KD

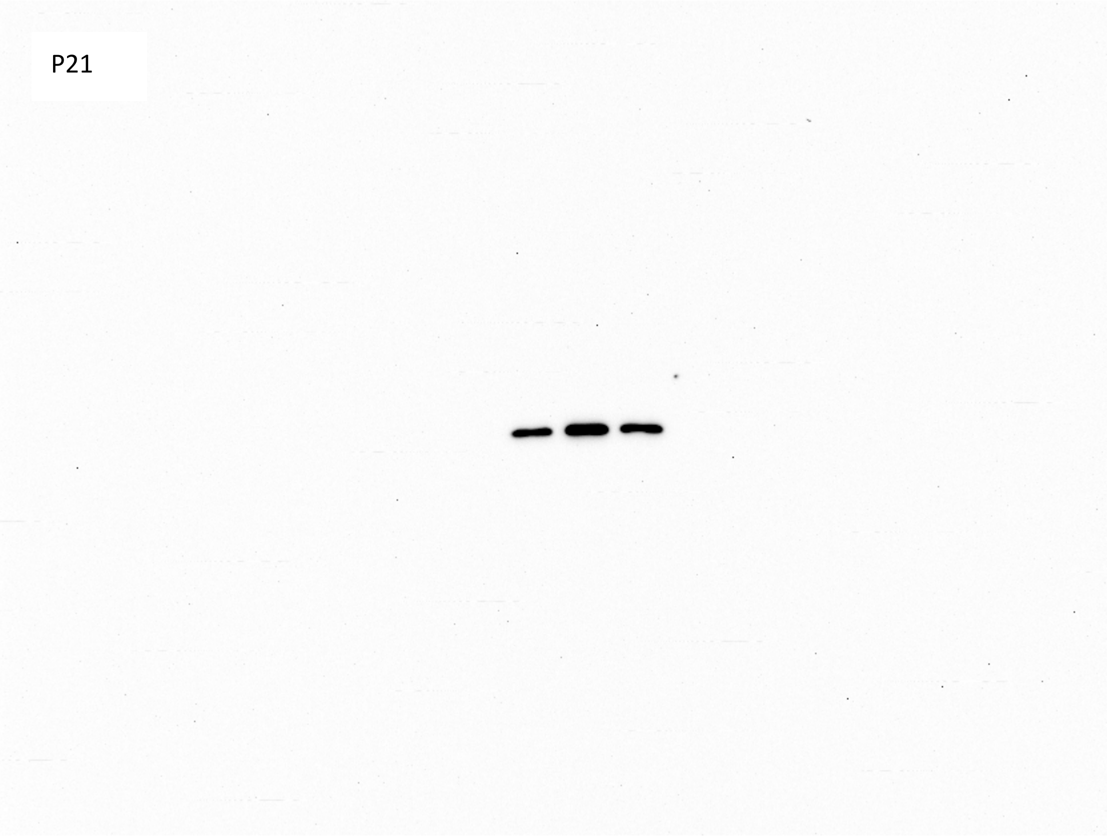
P21

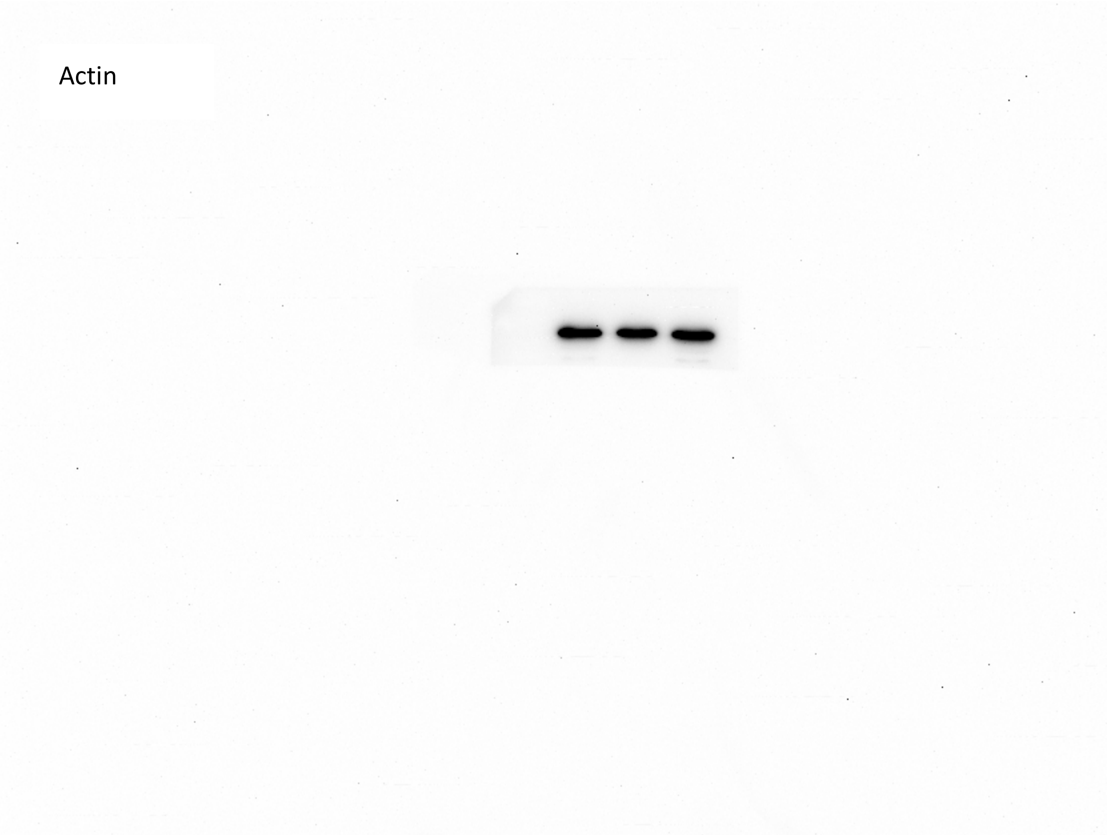
Actin

Fig4.(C). WB Original Data_Pin1_BRD4_NAP1L1_P21_Actin

Pin1

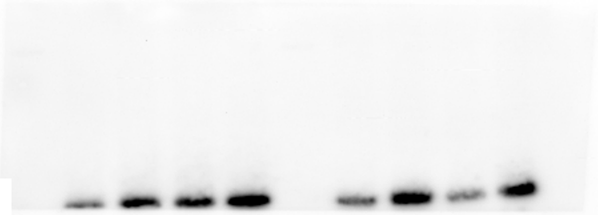

18KD

BRD4

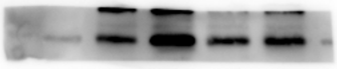

160KD

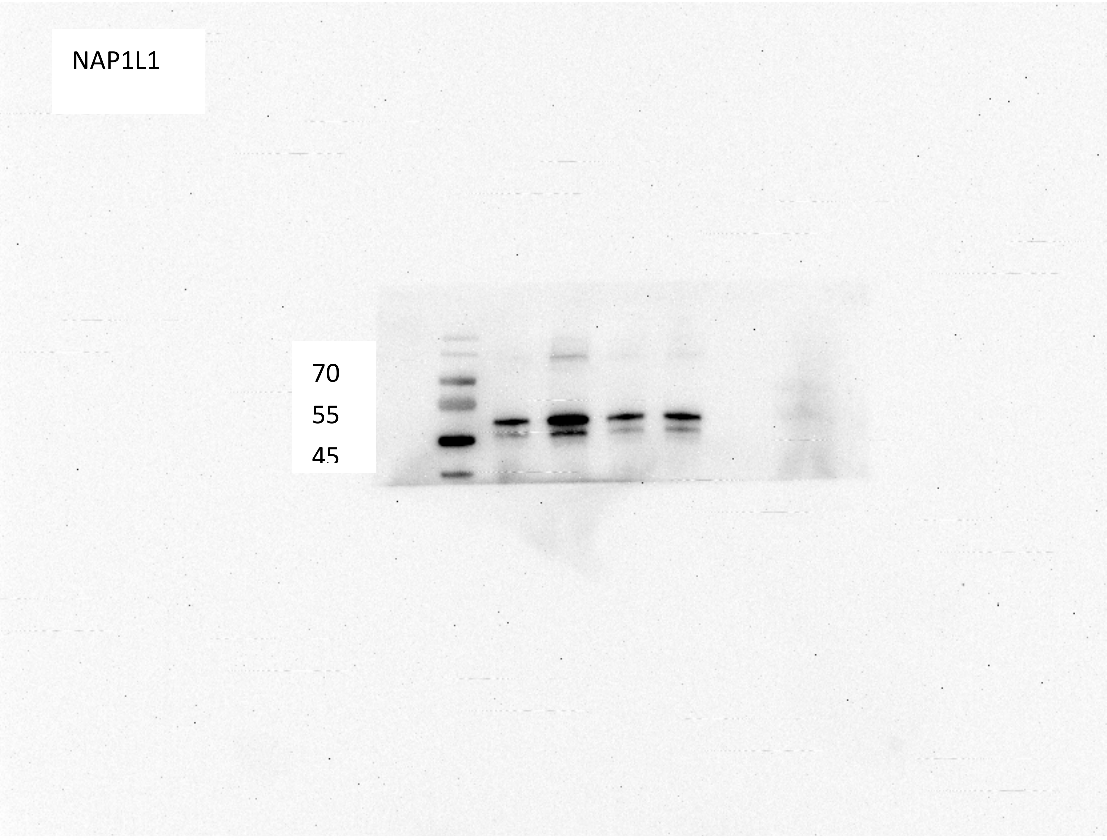
NAP1L1
70
55
45

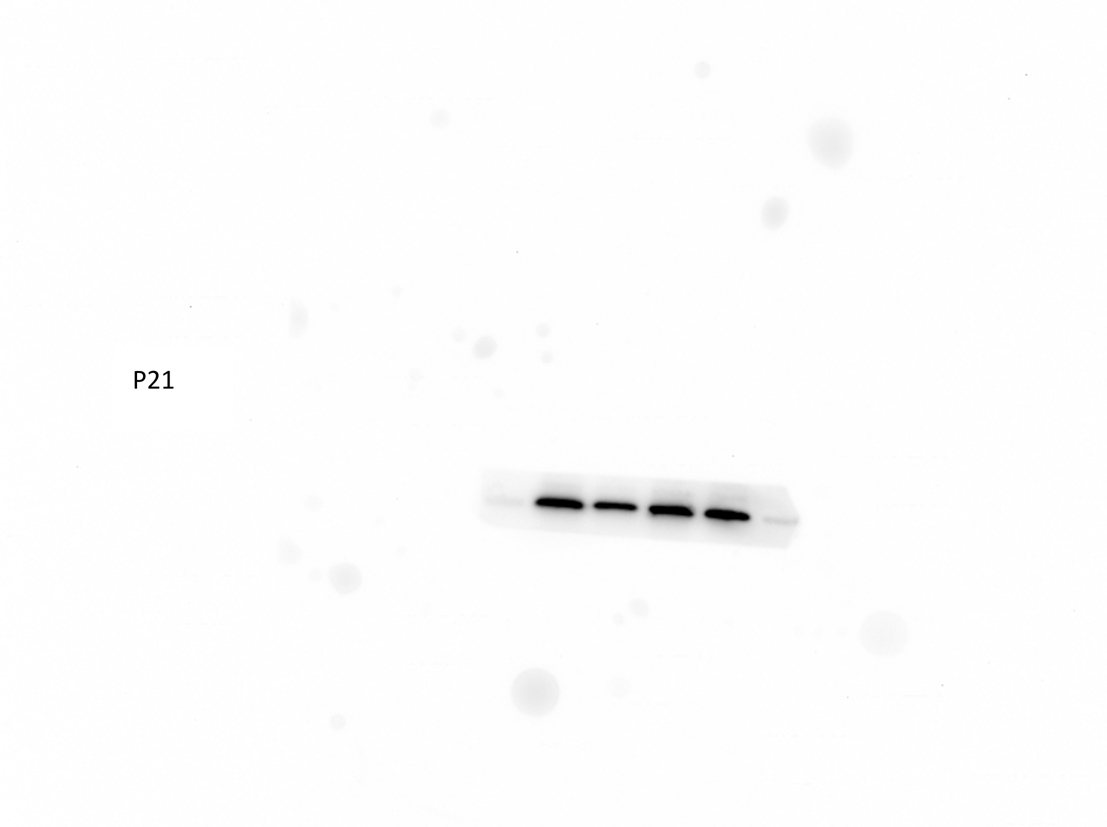
P21

Actin

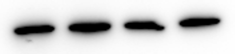

Supplement: Supplementary file 2 — Supplementary information of WB [file 41420_2022_1030_MOESM2_ESM.pdf]
