# Supplementary material for: Hyperglycemia induces gastric carcinoma proliferation and migration via the Pin1/BRD4 pathway
Source: Cell Death Discov. 2022 Apr 23;8:224. doi: 10.1038/s41420-022-01030-4 (PMC9035156; doi:10.1038/s41420-022-01030-4)
Supplement: Supplementary file 3 — Author Contribution Statement [file 41420_2022_1030_MOESM3_ESM.pdf]

Author Contribution Statement

Feng Peng: Conceptualization, Writing-Reviewing and Editing, Resources, Supervision, Funding acquisition.

Jianjian Yu: Conceptualization, Methodology, Investigation, Writing-Original Draft.

Dan Hu: Methodology,Investigation, Software.

Laicheng Wang and Zongcheng Fan: Writing-Reviewing and Editing.

Changsheng Xu: Methodology.

Yunchai Lin: Writing-Editing.

Xin Chen: Writing-Reviewing and Editing, Resources.

Jinxiu Lin: Writing-Reviewing and Editing, Resources, Funding acquisition.
